# Versatile Microgel Platform for Intervertebral Disc Degeneration Therapy: Targeting Pericyte‐Mediated Fibrosis and Protecting Nucleus Pulposus Cells

**DOI:** 10.1002/advs.77987

**Published:** 2026-09-30

**Authors:** Fei Ma, Zhen Zhao, Chuan Guo, Yuheng Liu, Yu Wang, Dengbo Yao, Weiqiang Lan, Dejun Zhong, Chen Fan, Yao He, Mei Zhang, Qingquan Kong, Yunfeng Lin

**Affiliations:** ^1^ Department of Orthopedic Surgery and Orthopedic Research Institute West China Hospital, Sichuan University Chengdu People's Republic of China; ^2^ Department of Orthopedics The Affiliated Hospital of Southwest Medical University Luzhou People's Republic of China; ^3^ Department of Orthopedics Surgery Hospital of Chengdu Office of People's Government of Tibetan Autonomous Region Chengdu People's Republic of China; ^4^ Macao Translational Medicine Center Macau University of Science and Technology Taipa People's Republic of China; ^5^ State Key Laboratory of Oral Diseases National Center for Stomatology National Clinical Research Center for Oral Diseases West China Hospital of Stomatology, Sichuan University Chengdu People's Republic of China

**Keywords:** fibrosis, intervertebral disc degeneration, microgel platform, nucleus pulposus cell protection, pericyte

## Abstract

Fibrosis is both a consequence and a driving factor of intervertebral disc degeneration (IVDD); however, the origins, pathways, and regulatory mechanisms of fibrotic effector cells remain incompletely understood, complicating antifibrotic therapy development. In this study, we identified pericytes as novel fibrotic effector cells in IVDD, contributing to fibrosis through activation of the TGF‐β signaling pathway. To address this, we developed a microgel platform (MMS@TRP) designed to specifically inhibit pericyte activation and pro‐fibrotic transition. MMS@TRP was constructed by integrating a tetrahedral framework nucleic acid (tFNA)‐based nanocarrier (TRP), which encapsulates a miR‐21 inhibitor and is conjugated with a pericyte‐targeting peptide (pPB), onto tannic acid (TA)‐based metal‐phenolic network (MPN)‐functionalized gelatin methacryloyl microspheres (GelMA MS). This microgel system protects TRP from enzymatic degradation by nucleases while facilitating its pH‐sensitive release. The early‐stage release of TRP from MMS@TRP ensures targeted delivery of the miR‐21 inhibitor to pericytes, suppressing pericyte proliferation, migration, and myofibroblast transition by blocking the TGF‐β signaling pathway. Simultaneously, sustained TA release provides prolonged protection to nucleus pulposus cells (NPCs) through reactive oxygen species (ROS) scavenging, mitochondrial preservation, and anti‐inflammatory effects. Collectively, the MMS@TRP platform presents a versatile and innovative approach for mitigating IVDD by inhibiting fibrosis and protecting NPCs.

## Graphical abstract

The engineered MMS@TRP microgel enables targeted, acid‐responsive delivery of a miR‐21 inhibitor to pericytes. It effectively suppresses disc fibrosis by inhibiting TGF‐β signaling while protecting nucleus pulposus cells via sustained, antioxidant tannic acid release. This platform presents an innovative dual‐action strategy for treating IVDD and related inflammatory diseases.

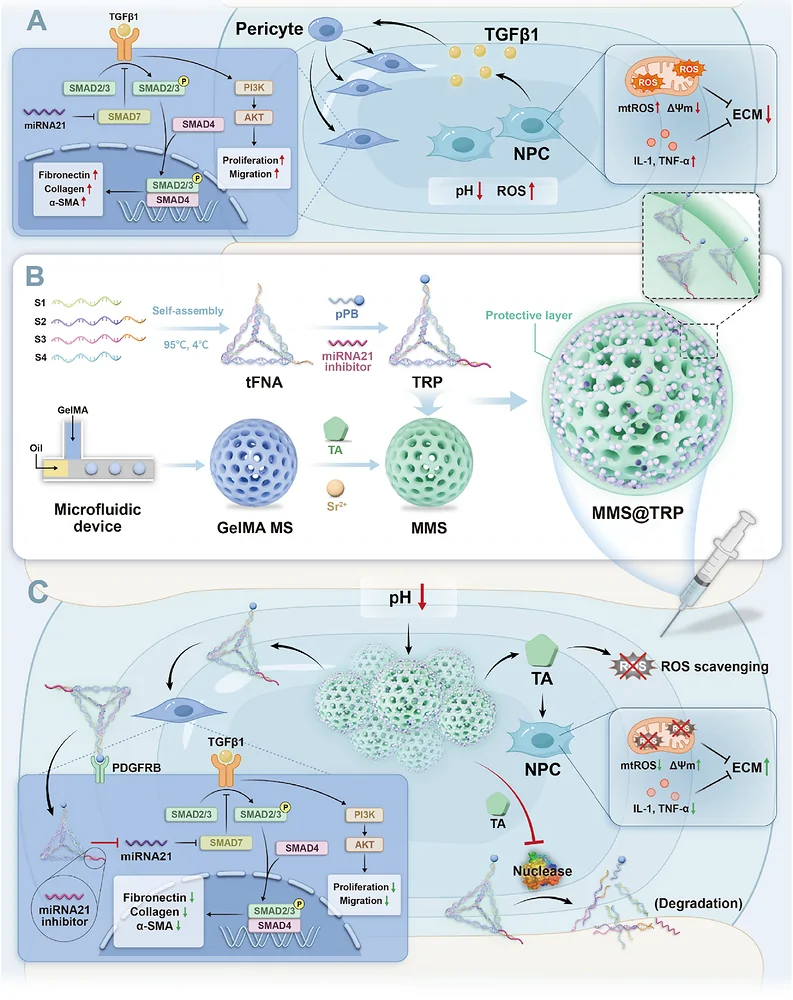

## 1 Introduction

Low back pain (LBP) is the leading cause of disability worldwide, with a particularly high prevalence among working‐age individuals, resulting in increased absenteeism, reduced productivity, and early retirement [1, 2]. By 2020, over 500 million individuals globally were affected by LBP, with projections indicating more than 800 million by 2050 [1, 2, 3]. Intervertebral disc degeneration (IVDD), the primary cause of LBP, has a radiographic prevalence rising from 16% at age 20 to 98% by age 70 [4]. IVDD is characterized by complex pathological manifestations, including progressive loss of resident cells, diminished functional activity and anabolic capacity, formation of an inflammatory microenvironment, accelerated extracellular matrix degradation, and aberrant vascularization accompanied by nerve ingrowth [5, 6]. These interconnected alterations establish a self‐perpetuating vicious cycle, driving pathological tissue remodeling of the disc. Specifically, IVDD‐related tissue remodeling involves breakdown of native matrix components and pathological fibrosis and/or calcification, collectively contributing to biomechanical failure of the functional spinal unit. Ultimately, these processes lead to disc narrowing, bulging, or herniation, causing nerve compression or irritation and back pain [7, 8, 9]. Thus, pathological fibrosis represents both a consequence and a driver of IVDD progression.

Within the degenerated intervertebral disc (IVD), myofibroblast transition of fibrotic effector cells results in the accumulation of fibrotic components such as collagen I and fibronectin [8]. Degenerated nucleus pulposus cells (NPCs) have been reported as the primary fibrotic effector cells in IVDD [10, 11, 12]. Factors such as inflammation, senescence, oxidative stress, and abnormal mechanical loading induce this phenotypic transition of NPCs in IVDD [12, 13, 14, 15]. Recent studies have also identified macrophages, PDGFA+ annulus fibrosus (AF) cells, and CD45+/COL1A1+ myeloid‐derived fibrocytes as additional effector cells contributing to pathological fibrosis in IVDD [16, 17]. However, other cell types, including mesenchymal stem cells (MSCs), epithelial‐mesenchymal transition (EMT) cells, and pericytes, have also been recognized as important sources of myofibroblasts in various fibrotic diseases [18, 19, 20]. Therefore, the origins and regulatory mechanisms underlying myofibroblast emergence in IVDD warrant further investigation.

In this study, by analyzing spatially distinct single‐cell RNA sequencing (scRNA‐seq) datasets, we observed an increased abundance of pericytes within degenerated IVD tissues, including the outer annulus fibrosus (OAF), inner annulus fibrosus (IAF), cartilaginous endplates (CE), and nucleus pulposus (NP). Subsequent bioinformatic analyses revealed pericyte activation, pro‐fibrotic phenotypic transitions, upregulation of fibrosis‐associated molecules (including Col1 and Fn1), and activation of relevant signaling pathways. These findings suggest that pericytes function as effector cells driving fibrotic remodeling in IVDD. The observed increase in pericyte abundance and their pro‐fibrotic role in IVDD were consistently validated in human degenerated IVD tissues and rat IVDD models. Further experiments demonstrated that degenerated NPCs promote pericyte proliferation, migration, and myofibroblast transition by activating the TGF‐β signaling pathway (including the canonical TGF‐β/Smad pathway and the non‐canonical PI3K/AKT pathway), thereby exacerbating pathological fibrosis in IVDD [21]. Collectively, this study provides the first evidence delineating the contribution of pericytes to pathological fibrosis in IVDD. Furthermore, the results indicate that targeting pericytes, particularly through inhibiting the TGF‐β signaling pathway to block myofibroblast transition, represents a promising therapeutic strategy for mitigating fibrosis in IVDD.

Therefore, we developed a microgel delivery platform focused on targeted pericyte intervention (Figure 1). The platform was initially engineered using a tetrahedral framework nucleic acid (tFNA) nanocarrier conjugated with the PDGFRB‐targeting peptide pPB, enabling selective delivery of a miR‐21 inhibitor to pericytes. The resulting tFNA complex (TRP) was subsequently loaded onto tannic acid (TA)‐based metal‐phenolic network (MPN)‐functionalized gelatin methacryloyl (GelMA) microspheres through hydrogen bonds, π–π stacking, and hydrophobic interactions, generating the microgel system MMS@TRP. Incorporation of the TA‐based MPN protected TRP from nuclease‐mediated enzymatic degradation and imparted stimuli‐responsive properties, allowing TRP release in the acidic microenvironment of degenerated IVDs. Both in vitro and in vivo experiments demonstrated efficient TRP internalization by pericytes via PDGFRB‐mediated endocytosis. Following internalization, the miR‐21 inhibitor effectively suppressed TGF‐β signaling, thereby inhibiting the pericyte‐to‐myofibroblast transition and fibrotic matrix synthesis. Meanwhile, sustained TA release provided long‐term protection for NPCs through reactive oxygen species (ROS) scavenging, mitochondrial preservation, and anti‐inflammatory effects. Collectively, the MMS@TRP platform represents a versatile and innovative strategy for alleviating IVDD by simultaneously suppressing pathological fibrosis and protecting NPCs. Furthermore, this platform provides a generalizable strategy for the protective delivery of framework nucleic acid nanocarriers and may be applicable to a broad range of inflammation‐related diseases.

**FIGURE 1:**
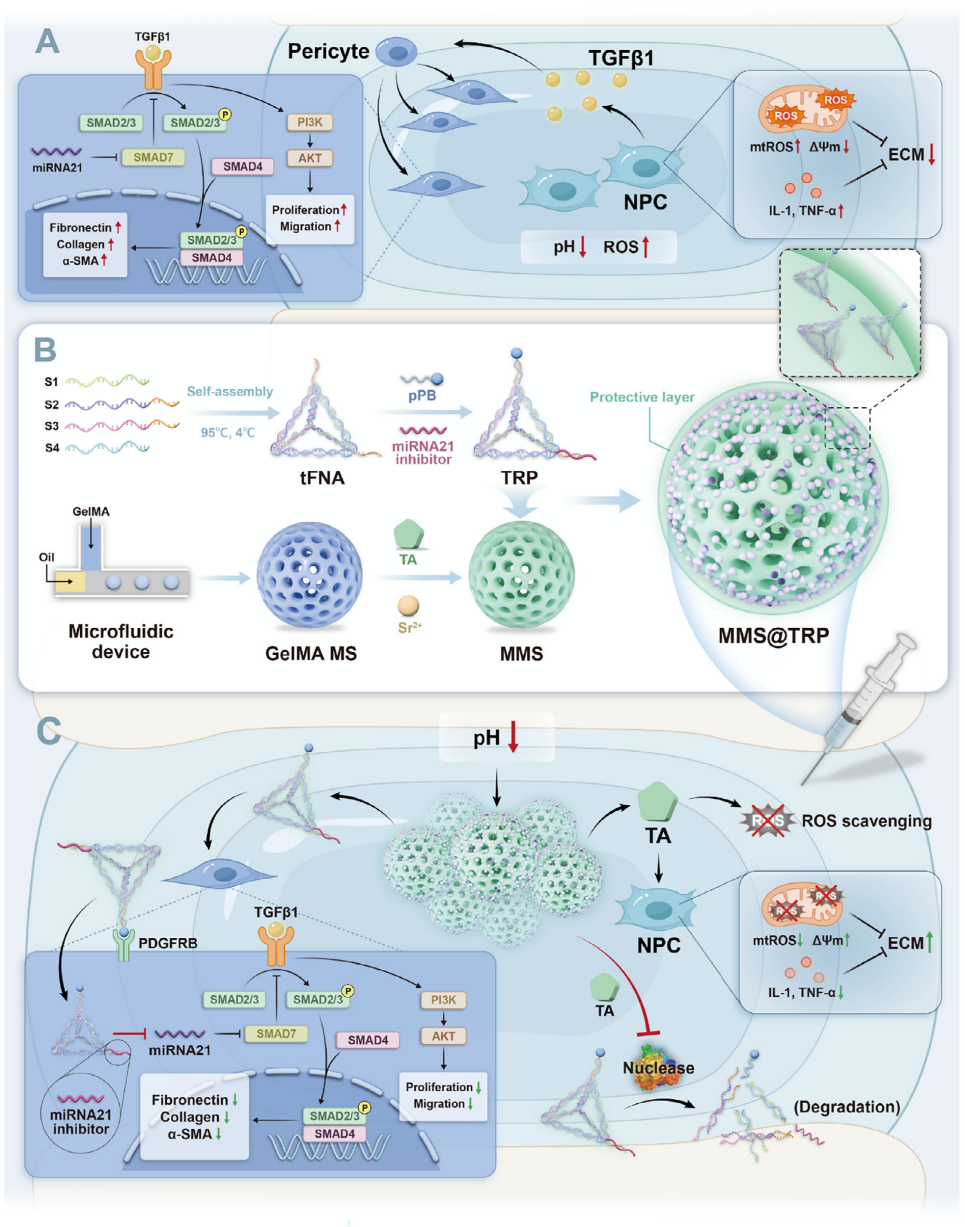
Schematic illustration of the pro‐fibrotic role of pericytes, the construction of MMS@TRP, and its intradiscal therapeutic mechanism. (A) In IVDD, degenerated NPCs promote pericyte activation and pro‐fibrotic transition through activation of the TGF‐β signaling pathway. (B) Schematic overview of TRP and MMS@TRP fabrication. (C) Following intradiscal injection, MMS@TRP releases TRP in the acidic microenvironment, thereby selectively inhibiting TGF‐β signaling in pericytes to exert anti‐fibrotic effects. Additionally, released TA protects NPCs through ROS scavenging, mitochondrial preservation, and anti‐inflammatory activity.

## 2 Results and Discussion

### 2.1 Pericytes Act as a Fibrosis Effector Cell in IVDD

scRNA‐seq analysis effectively delineates the multicellular ecosystem and molecular dynamics underlying IVDD, enabling the identification of disease‐driving cell populations and their dysregulated gene regulatory networks [22]. Although several scRNA‐seq studies have consistently reported increased pericyte abundance in degenerated IVDs, the role of pericytes in IVDD pathogenesis has not yet been investigated [16, 23, 24, 25].

In this study, a spatially distinct scRNA‐seq dataset of IVDs was obtained from the National Omics Data Encyclopedia (accession: OEP003834) [24]. Using established lineage‐specific marker genes, ten distinct cell populations were identified and visualized by UMAP dimensionality reduction (Figure 2A). These populations included chondrocytes, endothelial cells, pericytes, progenitor cells, fibroblasts, osteoprogenitor cells, notochordal cells, macrophages, lymphatic endothelial cells, and proliferating cells. Cluster‐specific marker genes for each cell type were identified and visualized in a dot plot (Figure 2B). Spatial distribution analysis revealed that, in healthy IVDs, pericytes were predominantly localized in the OAF (2.47%), with minimal distribution in the CE (0.196%). Pericytes were nearly absent in the IAF (0.059%) and NP (0.023%). In contrast, degenerated IVDs exhibited markedly increased pericyte abundance across all regions (OAF: 9.252%; CE: 4.593%; IAF: 6.94%; NP: 0.663%), indicating progressive infiltration from the outer to the inner regions of the IVD (Figure 2C). This pattern of pericyte accumulation throughout the disc was further validated in both human IVD tissues and a rat IVDD model. Immunofluorescence staining of mildly degenerated human IVDs (grade II) revealed only sparse RGS5+/PDGFRβ+ pericytes, whereas severely degenerated IVDs (grade V) exhibited abundant pericyte accumulation in the IAF and CE, with scattered distribution in the NP (Figure 2D). Similar findings were observed in the rat model (Figure 2E). The spatially distinct scRNA‐seq dataset and the observed redistribution of pericytes in human and rat IVD tissues during IVDD support a migration‐associated pattern. However, as these findings are derived from static analyses, future lineage‐tracing studies will be needed to determine the migratory trajectory of pericytes during IVDD progression.

**FIGURE 2:**
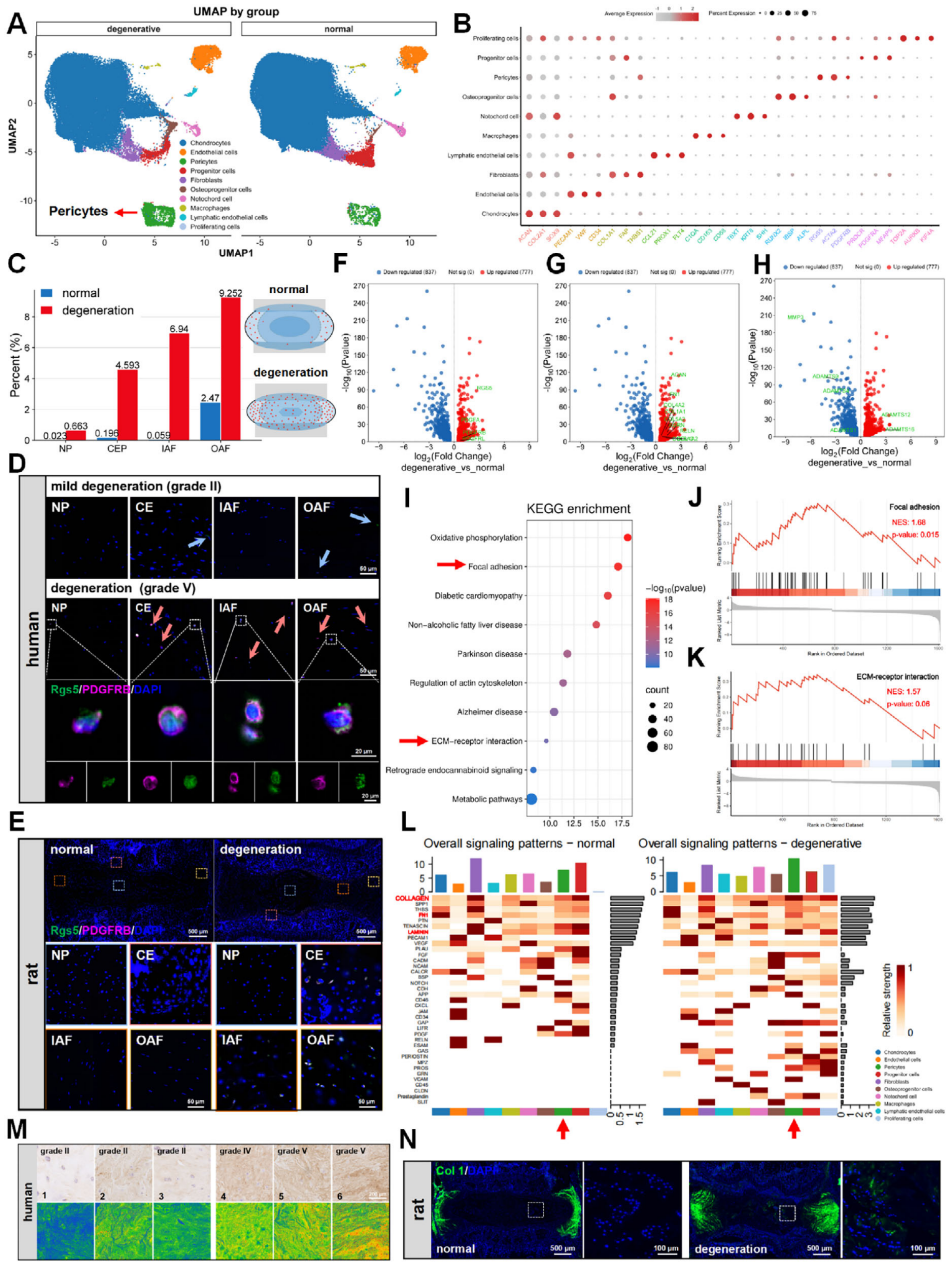
Pericytes function as fibrosis effector cells in IVDD. (A) UMAP dimensionality reduction visualization of distinct cell populations. (B) Dot plot depicting cluster‐specific marker gene expression. (C) Spatial distribution and quantification of pericyte proportions within the IVD. (D) Immunofluorescence staining analysis of RGS5+/PDGFRβ+ pericytes in mildly and severely degenerated human IVD tissues. (E) Immunofluorescence analysis of RGS5+/PDGFRβ+ pericytes in normal and degenerated rat IVD tissues. (F‐H) Volcano plots showing DEGs in pericytes. (I) KEGG enrichment analysis of pericyte‐associated DEGs. (J) GSEA analysis highlighting activation of the focal adhesion pathway in pericytes. (K) GSEA analysis identifying activation of the ECM‐receptor interaction pathway in pericytes. (L) Cell‐Chat analysis revealing ECM‐related intercellular signaling between pericytes and other cell populations. (M) Immunohistochemical comparison of Col1 expression levels in NP tissues from patients with mild versus severe IVDD. (N) Immunofluorescence staining demonstrating Col1 expression in normal and degenerated rat IVD tissues.

To further investigate the role of pericytes in IVDD, we performed bioinformatic analyses focused on pericyte populations. Volcano plot analysis revealed upregulation of genes associated with pericyte activation and/or proliferation, including RGS5, PDGFRB, and PDGFA (Figure 2F), as well as fibrosis‐associated ECM components, including COL1 and FN1 (Figure 2G), and laminin/integrin receptor family genes, including LAMB2, ITGB1, and ITGA1 (Figure S1A,B), in degenerated IVDs. In parallel, ECM‐degrading enzymes, including MMP3 and ADAMTS4, were downregulated (Figure 2H). These transcriptional alterations suggest pericyte activation and a pro‐fibrotic phenotypic transition during IVDD progression [26, 27, 28, 29, 30]. Kyoto Encyclopedia of Genes and Genomes (KEGG) enrichment analysis demonstrated that differentially expressed genes (DEGs) were significantly enriched in pathways associated with pro‐fibrotic phenotypic transition and ECM synthesis, including focal adhesion, ECM‐receptor interaction, and regulation of the actin cytoskeleton (Figure 2I). Gene Set Enrichment Analysis (GSEA) further confirmed activation of the focal adhesion and ECM‐receptor interaction pathways in pericytes (Figure 2J,K). Cell‐Chat analysis revealed enhanced intercellular communication involving ECM‐related signaling patterns, including COLLAGEN, FN1, and LAMININ, between pericytes and other cell populations (Figure 2L). Collectively, these findings indicate pronounced pericyte activation and pro‐fibrotic transition during IVDD progression. Fibrotic remodeling in IVDD was further validated by immunohistochemical analysis of human NP tissues and immunofluorescence staining in the rat model (Figure 2M,N; Figure S1C). As shown in Figure 2D,E,M,N, both pericyte accumulation within IVDs and the extent of fibrotic remodeling progressively increased with IVDD severity.

Multipotent pericytes have emerged as key effector cells in fibrosis‐associated diseases, such as pulmonary fibrosis, renal fibrosis, and spinal cord injury‐induced scarring [28, 31, 32, 33]. While the data described above suggest pericyte activation and a shift toward a pro‐fibrotic phenotype during IVDD, further evidence is required to confirm their role as effectors of fibrosis in IVDD pathogenesis. To address this, pericytes were transplanted into a rat IVDD model one week after induction. At four weeks post‐transplantation, immunofluorescence staining revealed significantly higher numbers of RGS5+/PDGFRβ+ pericytes in the transplantation group compared to the model‐only group (Figure 3A,B). Hematoxylin and eosin (H&E) staining indicated NP atrophy and blurred boundaries between NP and AF regions in both model‐only and transplantation groups (Figure 3C). Safranin O/Fast Green staining demonstrated greater proteoglycan loss and collagen deposition in discs from the pericyte transplantation group (Figure 3C). Additionally, immunofluorescence analysis revealed significantly higher Col1 levels in the transplantation group compared to the model‐only group (Figure 3D; Figure S1D). These findings collectively confirm that pericytes play a significant role as effector cells driving fibrosis in IVDD.

**FIGURE 3:**
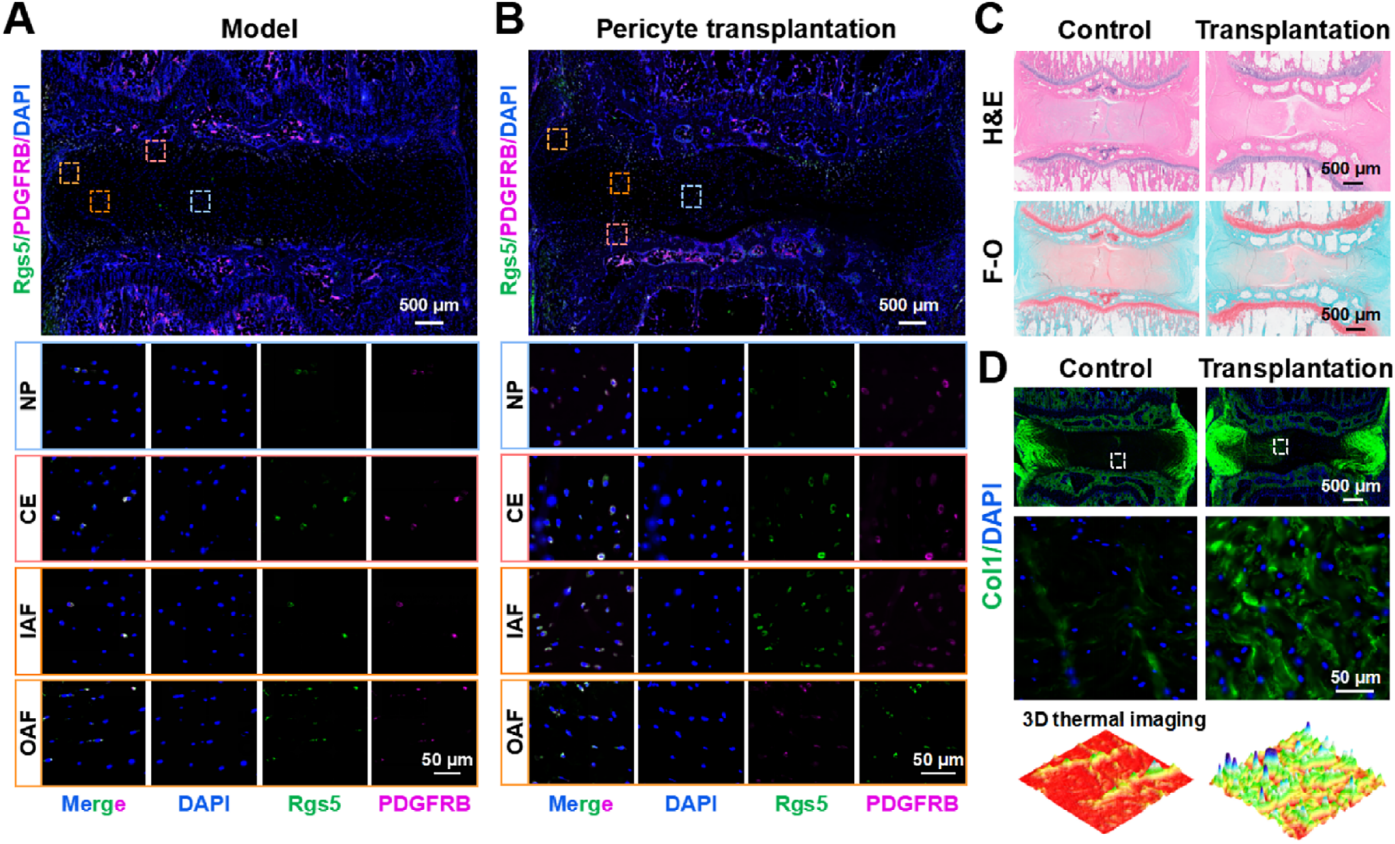
Pericyte transplantation accelerates fibrotic remodeling in IVDD. (A, B) Immunofluorescence staining comparing RGS5+/PDGFRβ+ pericyte distribution across disc regions (NP, CE, IAF, and OAF) in the model‐only and pericyte transplantation groups. (C) Representative images of H&E and Safranin O‐Fast Green staining comparing the model and transplantation groups. (D) Immunofluorescence staining and 3D thermal imaging analyses comparing Col1 expression in IVD tissues from the model‐only and pericyte transplantation groups.

### 2.2 Degenerated NPCs Promote Pericyte Activation and Myofibroblast Transition via the TGF‐β Signaling Pathway

NPCs constitute the most abundant and functionally significant cellular population within the IVD. NPC degeneration is an early event in IVDD pathogenesis, influencing other cell types by secreting cytokines such as ROS, IL‐1β, IL‐6, TGFβ1, VEGFA, NGF, and substance P [34, 35, 36, 37]. This process exacerbates degeneration through additional NPC damage, immune cell infiltration, and vascular or neuronal invasion [36]. We hypothesized that pericyte proliferation, inward migration, and transition into myofibroblasts during IVDD are associated with factors secreted by degenerated NPCs (dNPCs). To validate this hypothesis, pericytes were co‐cultured with conditioned medium from TBHP‐induced dNPCs, simulating the IVDD microenvironment. CCK‐8 assays (Figure S2A), migration assays (Figure 4B), and immunofluorescence detection of myofibroblast markers (α‐SMA and Col1) (Figure 4C; Figure S2B) demonstrated that factors derived from dNPCs significantly promoted pericyte proliferation, migration, and myofibroblast differentiation. These findings provide preliminary evidence that dNPCs contribute to pericyte activation and subsequent fibrosis during IVDD progression.

**FIGURE 4:**
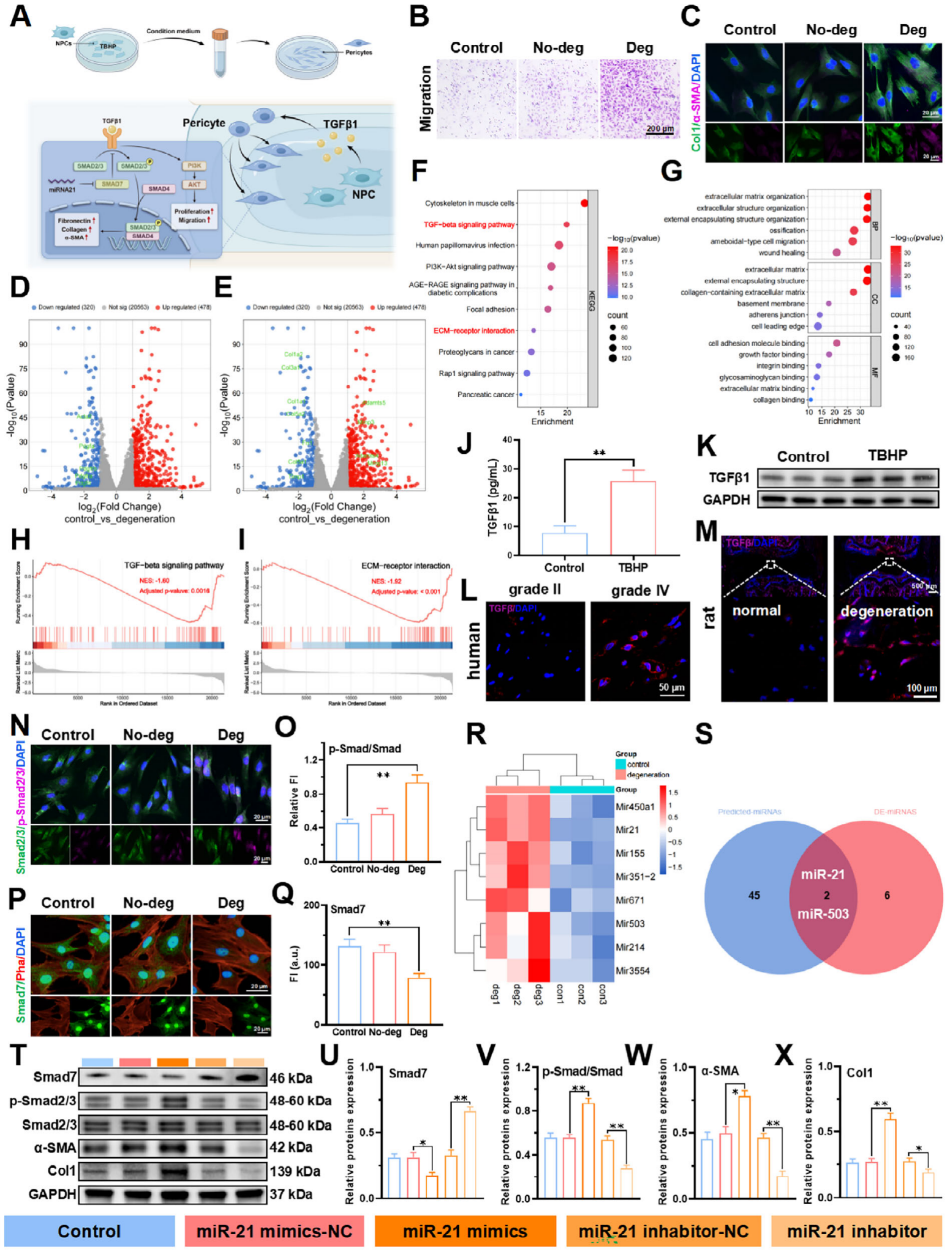
Mechanistic insights into pericyte‐driven fibrosis in IVDD. (A) Schematic illustrating mechanisms underlying pericyte proliferation, migration, and pro‐fibrotic transformation in IVDD. (B) Transwell assay demonstrating enhanced pericyte migration in response to dNPC‐conditioned medium. (C) Immunofluorescence analysis showing increased expression of fibrotic markers Col1 and α‐SMA in pericytes after dNPC treatment. (D, E) Volcano plots identifying DEGs associated with pericyte activation and myofibroblast differentiation. (F) KEGG enrichment analysis highlighting significant pathways associated with DEGs. (G) GO enrichment analysis of DEGs illustrating relevant biological processes, cellular components, and molecular functions. (H, I) GSEA confirming activation of TGF‐β signaling and ECM‐receptor interaction pathways in pericytes treated with dNPC‐conditioned medium. (J) ELISA quantification of TGFβ1 levels in normal and TBHP‐treated NPCs (n = 3). (K) Western blot analysis confirming elevated TGFβ1 in TBHP‐treated NPCs. (L) Immunofluorescence staining comparing TGFβ1 expression in NP tissues from mildly versus severely degenerated human IVD samples. (M) Immunofluorescence staining demonstrating TGFβ1 expression in normal versus degenerated rat IVD tissues. (N, O) Immunofluorescence and quantitative analyses indicating increased Smad2/3 phosphorylation in pericytes treated with dNPC‐conditioned medium (n = 3). (P, Q) Immunofluorescence and quantitative analyses showing decreased Smad7 levels in pericytes after dNPC treatment (n = 3). (R) Heatmap illustrating miRNAs upregulated in pericytes following dNPC treatment. (S) Venn diagram identifying overlap between predicted and experimentally upregulated miRNAs. (T‐X) Western blot and quantitative analyses illustrating the effects of miR‐21 mimics and inhibitors on Smad7, p‐Smad2/3, Smad2/3, α‐SMA, and Col1 expression in pericytes (n = 3). Quantitative data are presented as mean ± SD. Error bars represent SD. Statistical analysis was performed using an unpaired two‐tailed Student's t‐test for panel (J) and one‐way ANOVA followed by Tukey's multiple‐comparisons test for panels (O, Q, U‐X). ^*^ *p* < 0.05, ^**^ *p* < 0.01.

To further investigate the underlying molecular mechanisms, RNA sequencing (RNA‐seq) was conducted. Principal component analysis (PCA) and heatmap clustering confirmed distinct transcriptional profiles in pericytes treated with dNPCs (Figure S2C,D). Compared to controls, the dNPC‐treated group exhibited 320 upregulated genes and 478 downregulated genes (Figure 4D,E). Volcano plot analysis showed significant upregulation of pericyte activation markers (PDGFRB, PDGFA), myofibroblast markers, and fibrosis‐associated genes (α‐SMA, Col1, Col3, Fn1, Timp1), along with downregulation of ECM‐degrading enzymes (MMP3, ADAMTS5) in pericytes following dNPC treatment (Figure 4D,E). KEGG enrichment analysis indicated that the DEGs were associated with several biological pathways, including cytoskeletal regulation in muscle cells, TGF‐β signaling, PI3K‐Akt signaling, focal adhesion, ECM‐receptor interaction, and Rap1 signaling pathways (Figure 4F). Gene Ontology (GO) enrichment analysis demonstrated significant involvement of DEGs in biological processes (BP) such as ECM organization and extracellular structural organization, cellular components (CC) such as ECM and basement membrane, and molecular functions (MF) including cell adhesion molecule binding, growth factor binding, and collagen binding (Figure 4G). GSEA further validated the activation of the TGF‐β signaling pathway, ECM‐receptor interaction, and PI3K‐Akt signaling pathway in pericytes exposed to dNPC‐conditioned medium (Figure 4H,I; Figure S2E). These pathways are well‐documented to be crucial for pericyte proliferation, migration, and myofibroblast transformation [38, 39, 40].

The collective activation of these critical signaling pathways suggests that TGFβ1 derived from dNPCs functions as a key initiating cytokine. Specifically, TGFβ1 activates the canonical TGFβ/Smad signaling pathway, enhances Smad2/3 phosphorylation, and consequently upregulates expression of α‐SMA, Fn1, and Col1, driving the pericyte‐to‐myofibroblast transition [39]. Concurrently, TGFβ1 also activates non‐canonical PI3K‐Akt/MAPK signaling pathways, promoting pericyte proliferation and migration [38, 40]. Indeed, significantly elevated TGFβ1 levels were detected in dNPCs through ELISA and western blot analyses (Figure 4J,K; Figure S2F). Immunofluorescence staining further confirmed substantially higher TGFβ1 expression in severely degenerated human NP tissues compared to mildly degenerated samples (Figure 4L; Figure S2G). Similar results were obtained in a rat IVDD model (Figure 4M; Figure S2H). Immunofluorescence evaluation of canonical TGFβ signaling confirmed significantly elevated Smad2/3 phosphorylation and reduced expression of Smad7, a critical negative regulator, in pericytes treated with dNPC‐conditioned medium (Figure 4N–Q). These results collectively indicate robust activation of the TGFβ/Smad pathway in pericytes following exposure to dNPC‐derived factors [41]. Additionally, immunofluorescence analysis of the TGFβ1 antibody neutralization experiment showed that TGFβ1 blocking significantly inhibited the promotive effect of the dNPC‐derived supernatant on the myofibroblast transition of pericytes (Figure S2I). To further elucidate the causal role of TGFβ1 in this process, gain‐ and loss‐of‐function approaches were performed using recombinant TGFβ1 stimulation and pharmacological inhibition of the TGFβ type I receptor using SB‐431542[]. Western blot analysis revealed that both recombinant TGFβ1 and dNPC‐conditioned medium markedly enhanced Smad2/3 phosphorylation, increased the expression of fibrotic markers Col1 and α‐SMA, and decreased Smad7 expression in pericytes (Figure S3A–J). Conversely, SB‐431542 treatment significantly attenuated the pericyte‐to‐myofibroblast transition induced by both recombinant TGFβ1 and dNPC‐conditioned medium, with a more pronounced inhibitory effect observed in recombinant TGFβ1‐stimulated pericytes (Figure S3A–J). Collectively, our findings indicate that dNPCs primarily activate the TGF‐β signaling pathway in pericytes via TGFβ1, promoting their proliferation, migration, and transition to myofibroblasts. This mechanism closely resembles the established role of elevated VEGFA expression in promoting vascular invasion in IVDD [24]. However, dNPCs exhibit increased expression of cytokines such as matrix metalloproteinases (MMPs) and ROS, which may contribute to pericyte activation and fibrotic progression through TGF‐β‐dependent or other signaling pathways [35]. This explains why TGF‐β1 blockade failed to completely inhibit dNPC‐induced transition of pericytes into myofibroblasts, despite the dominant role of dNPC‐derived TGF‐β1.

To further investigate the contribution of PI3K‐Akt signaling to pericyte activation, preliminary validation experiments were performed. Consistent with the RNA‐seq analysis, western blotting demonstrated that dNPC‐conditioned medium significantly activated PI3K‐Akt signaling in pericytes, as evidenced by increased phosphorylation of Akt. Pharmacological inhibition of PI3K using LY294002 effectively reduced Akt phosphorylation, whereas the expression levels of fibrotic markers Col1 and α‐SMA were only moderately decreased (Figure S4A,B). Functional assays further revealed that PI3K inhibition partially suppressed dNPC‐induced pericyte proliferation and migration (Figure S4C,D). These results suggest that PI3K‐Akt signaling contributes partially to the proliferation and migration of pericytes, while exerting a relatively limited effect on the pericyte‐to‐myofibroblast transition. Although PI3K‐Akt signaling represents an important non‐canonical pathway associated with TGFβ signaling, it can also be activated by diverse stimuli and signaling networks [42, 43]. Therefore, the precise mechanisms underlying PI3K‐Akt activation and its regulation of pericyte behavior during IVDD progression require further investigation.

### 2.3 miR‐21 Inhibitor Suppresses Pro‐Fibrotic Transition of Pericytes by Upregulating Smad7 and Blocking TGF‐β Signaling Pathway

TGFβ1 exerts a dual role in IVDD: while essential for maintaining intrinsic disc cell function, such as preserving ECM homeostasis in healthy NPCs, it also promotes IVDD progression through processes like fibrosis and ossification [35, 44, 45]. Consequently, directly antagonizing TGFβ1, for example by using TGFβ1 antibodies or receptor blockers, may cause adverse effects, underscoring the need for more precise therapeutic interventions. Smad7, a crucial inhibitory regulator of the TGF‐β signaling pathway, is known to be downregulated in pericytes during fibrosis‐related diseases [46]. Consistent with these findings, we observed reduced Smad7 expression in pericytes from degenerated human and rat IVD tissues (Figure S5A,B). Previous studies have demonstrated that targeted upregulation of Smad7 effectively inhibits the TGF‐β pathway, suppressing the pro‐fibrotic phenotypic transition of pericytes [47, 48]. Therefore, selective modulation of Smad7 in pericytes represents a promising therapeutic strategy for IVDD‐related fibrosis. MicroRNAs (miRNAs) regulate gene expression and have been implicated as critical factors in IVDD pathogenesis, making them attractive candidates for biomarkers and therapeutic interventions [49]. To identify miRNAs targeting Smad7 specifically in pericytes, we used miRDB, TargetScan, and miRTargetLink, predicting 47 candidate miRNAs (Figure S5C). Concurrently, RNA‐seq analysis identified eight miRNAs upregulated in pericytes following dNPC treatment (Figure 4R). Integrating these datasets highlighted miR‐21 and miR‐503 as potential regulators (Figure 4S). miR‐21 is significantly elevated in various fibrotic conditions, such as spinal cord injury, renal fibrosis, and lung fibrosis, and promotes fibrosis by inhibiting Smad7 [50, 51, 52]. In this study, dual‐luciferase reporter assays further demonstrated that miR‐21 directly binds to the 3′‐UTR of Smad7, thereby suppressing its expression (Figure S6A–C). These findings, combined with previous evidence that Smad7 upregulation mitigates fibrosis, support using miR‐21 inhibition as an anti‐fibrotic therapeutic strategy for IVDD. Additionally, our prior studies confirmed the protective effects of miR‐21 inhibitors on NPCs [53, 54]. Therefore, the miR‐21 inhibitor was selected to specifically increase Smad7 expression in pericytes while avoiding negative impacts on NPCs.

To validate the effects of miR‐21 inhibition in pericytes, western blot analysis was conducted to measure key proteins involved in the myofibroblast transition. Transfection with miR‐21 mimics significantly reduced Smad7 expression, accompanied by increased phosphorylation of Smad2/3 and elevated expression of α‐SMA and Col1 (Figure 4T–X). Conversely, miR‐21 inhibitor treatment significantly elevated Smad7 levels and decreased p‐Smad2/3, α‐SMA, and Col1 expression (Figure 4T–X). Furthermore, Smad7 knockdown using siRNA significantly abolished the anti‐fibrotic effects induced by miR‐21 inhibition, as evidenced by restored Smad2/3 phosphorylation and increased expression of α‐SMA and Col1 (Figure S7A–E). These results indicate that miR‐21 inhibition effectively suppresses the pro‐fibrotic transition of pericytes through Smad7 upregulation and subsequent blockade of TGF‐β signaling. Thus, the miR‐21 inhibitor holds therapeutic potential for reducing fibrosis in IVDD.

### 2.4 Construction and Characterization of the Pericyte‐Targeting Nanocarrier TRP

Achieving targeted delivery of the miR‐21 inhibitor to pericytes within IVDs is critical for effective anti‐fibrotic therapy. Due to the dense structural organization of IVD tissue, the limited cellular uptake, and rapid systemic clearance of unmodified small RNAs, an efficient pericyte‐targeted nanocarrier capable of enhanced tissue penetration is required. Tetrahedral framework nucleic acids (tFNAs), DNA‐based nanostructures known for efficient cellular internalization, have shown promise as effective carriers for small RNA delivery [55, 56, 57]. Furthermore, tFNAs conjugated with targeting peptides have demonstrated improved penetration into cartilage tissues for therapeutic agent delivery [58]. The cyclic peptide CSRNLIDC (pPB), derived from the receptor‐binding domain of the PDGF‐B chain, specifically binds to the pericyte marker PDGFRB with high affinity without activating downstream proliferative signaling [59]. Therefore, we developed a novel tFNA complex (TRP) functionalized with the peptide pPB to enable pericyte‐targeted delivery of the miR‐21 inhibitor (Figure 5A).

**FIGURE 5:**
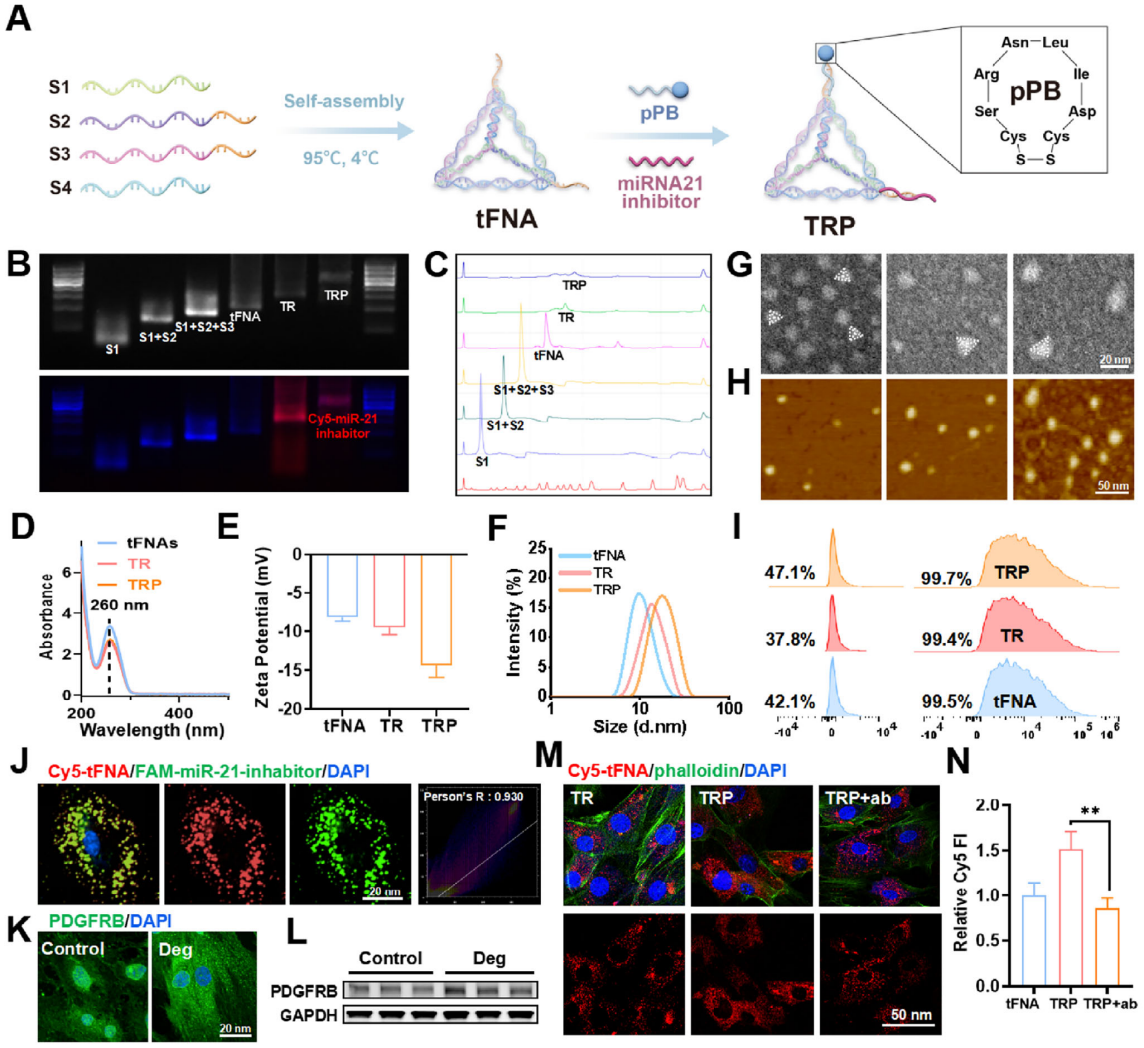
Construction and characterization of TRP. (A) Schematic illustration of the TRP preparation process. (B) PAGE confirming successful TRP formation. (C) HPCE analysis verifying TRP synthesis. (D) UV–vis spectra of tFNAs, TR, and TRP. (E) Zeta potential analysis of tFNAs, TR, and TRP measured by DLS (n = 3). (F) DLS measurement showing particle size distributions of tFNAs, TR, and TRP. (G) TEM images of tFNAs, TR, and TRP. (H) AFM images of tFNAs, TR, and TRP. (I) Flow cytometry quantification of Cy5‐labeled tFNAs, TR, and TRP uptake by pericytes at 4 and 8 h. (J) Fluorescence colocalization analysis of Cy5‐tFNAs and FAM‐miR‐21 inhibitor in pericytes following TRP uptake. (K) Immunofluorescence analysis comparing PDGFRB expression in control and dNPC‐treated pericytes. (L) Western blot analysis comparing PDGFRB expression in control versus dNPC‐treated pericytes. (M, N) Confocal microscopy and quantitative fluorescence analysis of TR and TRP uptake in dNPC‐treated pericytes, assessing the blocking effect of anti‐PDGFRB antibodies on TRP internalization (n = 3). Quantitative data are presented as mean ± SD. Error bars represent SD. Statistical analysis was performed using one‐way ANOVA followed by Tukey's multiple‐comparisons test for panels (N). ^**^ *p* < 0.01.

First, two distinct sticky ends were engineered onto the S2 (S2‐P) and S3 (S3‐R) strands of the tFNAs, allowing hybridization via complementary base pairing with the sticky ends of peptide pPB and the miR‐21 inhibitor strand, respectively. This approach facilitated simultaneous incorporation of the pericyte‐targeting peptide and loading of the miR‐21 inhibitor onto tFNAs. Polyacrylamide gel electrophoresis (PAGE) and high‐performance capillary electrophoresis (HPCE) confirmed successful formation of miR‐21 inhibitor‐loaded tFNAs (TR) and co‐loaded tFNAs with miR‐21 inhibitor and pPB peptide (TRP), indicated by increased molecular weights after miR‐21 inhibitor incorporation and pPB conjugation (Figure 5B,C). Fluorescence colocalization of GelBlue and Cy5‐labeled miR‐21 inhibitor further verified the successful synthesis of TR and TRP (Figure 5B). Ultraviolet‐visible (UV–vis) spectroscopy confirmed nucleic acid‐specific absorbance peaks at 260 nm for tFNAs, TR, and TRP (Figure 5D). Incorporation of the negatively charged miR‐21 inhibitor and peptide pPB altered the zeta potentials of tFNAs, decreasing from ‐8.14 mV for tFNAs alone to ‐9.48 mV for TR and ‐14.32 mV for TRP (Figure 5E). Dynamic light scattering (DLS) measurements indicated hydrodynamic diameters of 9.7 nm (tFNAs), 13.1 nm (TR), and 17.7 nm (TRP) (Figure 5F). Transmission electron microscopy (TEM) and atomic force microscopy (AFM) imaging confirmed that tFNAs, TR, and TRP maintained similar tetrahedral structures following miR‐21 inhibitor loading and pPB conjugation, and corroborated the DLS size measurements (Figure 5G,H).

Based on biocompatibility evaluations with NPCs and Smad7 upregulation efficiency in pericytes, a TRP concentration of 200 nm was selected for subsequent cellular experiments (Figure S3A–C). Flow cytometry analysis revealed significantly enhanced uptake of TRP by pericytes (47.0%) compared to tFNAs (42.1%) and TR (37.8%) at 4 h; all groups reached comparable uptake levels by 8 h (Figure 5I). Furthermore, colocalization analysis using FAM‐miR‐21 inhibitor and Cy5‐tFNAs demonstrated a high degree of colocalization (Pearson's R = 0.930), confirming synchronized cellular internalization of both components and successful TRP synthesis (Figure 5J). Antibody blocking assays were conducted to assess PDGFRB‐mediated internalization of TRP. PDGFRB, a pericyte‐specific receptor, was significantly upregulated in pericytes from degenerated IVDs and following dNPC treatment (Figures 2F and 4D and 5K,L; Figure S2I,J; Figure S3D,E). Pericytes exposed to dNPC‐conditioned medium displayed markedly increased TRP uptake compared to TR alone. However, the enhanced uptake of TRP was effectively inhibited by anti‐PDGFRB antibody co‐treatment (Figure 5M,N). To further verify the pericyte‐targeting capability of TRP, we performed an in vitro comparative uptake assay in pericytes, NPCs, and macrophages. NPCs represent a major resident functional cell population within the IVD, whereas macrophages are key immune cells involved in the IVDD inflammatory microenvironment. Quantitative fluorescence analysis demonstrated significantly greater intracellular accumulation of TRP in pericytes than in NPCs and macrophages (Figure S9A–C). These findings indicate that TRP specifically targets pericytes via PDGFRB, promoting efficient cellular internalization. Immunofluorescence staining further confirmed that TRP treatment significantly upregulated Smad7, reduced Smad2/3 phosphorylation, and downregulated the myofibroblast markers Col1 and α‐SMA in pericytes (Figure S8F–K). Collectively, these results demonstrate that the engineered pPB‐functionalized nanocarrier TRP successfully delivers the miR‐21 inhibitor to pericytes, effectively suppressing their fibrotic phenotype transition.

### 2.5 Construction and Characterization of the MMS@TRP Microgel System

Because the IVD is avascular, therapeutic interventions typically rely on localized injection. However, given the deep‐tissue nature of the IVD and the potential for puncture‐induced degeneration, ideal therapeutic agents for IVDD must maintain prolonged efficacy following a single injection. Although the TRP complex exhibits efficient pericyte uptake, its inherent rapid degradation in vivo limits its direct clinical use [60]. Additionally, the inflammatory microenvironment of IVDD, characterized by elevated ROS levels, low pH, and increased pro‐inflammatory cytokines (e.g., IL‐1β, IL‐6, TNF‐α), results in oxidative stress, inflammation, ECM imbalance, and ultimately NPC senescence, dysfunction, and apoptosis [61]. Therefore, an optimal delivery system should enable sustained TRP release to target pericytes effectively while simultaneously protecting NPCs. Gelatin methacryloyl (GelMA) microspheres (MS) feature an ECM‐like architecture and superior water‐retention capacity, promoting NPC adhesion and proliferation, making them promising candidates for IVDD treatment [62]. Moreover, their intrinsic porous structure and compatibility with surface modification allow integration with nanomaterials, facilitating the development of multifunctional delivery platforms [63]. Polyphenols can coordinate with metal ions (e.g., Mn, Cu, Gd, Ti, Fe, Sr) to form supramolecular self‐assembled metal‐polyphenol networks (MPNs), which display strong universal surface adhesion and pH‐responsive release characteristics, rendering them highly effective as responsive coatings [64, 65, 66, 67, 68]. Additionally, polyphenols containing phenolic hydroxyl groups interact strongly with nucleic acids via hydrogen bonding, π‐π stacking, and hydrophobic interactions [54]. Thus, GelMA MS functionalized with MPNs can stably immobilize TRP on their surfaces, forming a pH‐responsive microgel delivery system. TA has demonstrated significant protective properties for NPCs, including ROS scavenging, mitochondrial preservation, and anti‐inflammatory action [53, 69, 70]. Sr^2^^+^ has also been reported to promote cartilage matrix synthesis, while TA/Sr^2^^+^‐based MPNs exhibit favorable cytocompatibility and cartilage‐protective effects [68]. Therefore, this study utilized GelMA MS functionalized with TA/Sr^2+^‐based MPNs as the foundational biomaterial to load TRP, establishing the multifunctional MMS@TRP microgel platform (Figure 6A).

**FIGURE 6:**
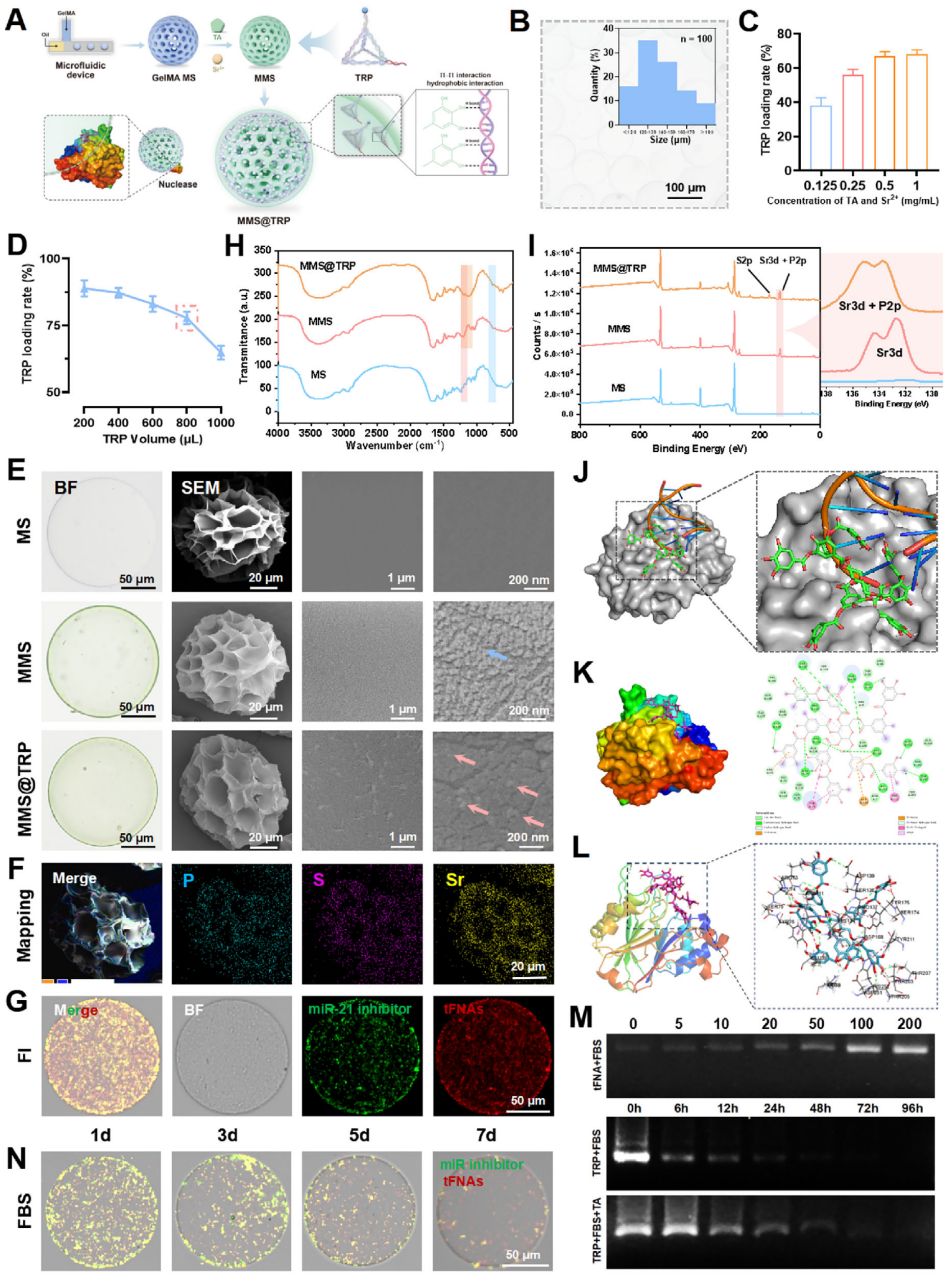
Construction and characterization of MMS@TRP. (A) Schematic illustration of the MMS@TRP preparation process. (B) Optical microscopy images and size distribution analysis of GelMA MS. (C) Influence of TA/Sr^2+^ concentration during MMS preparation on TRP loading efficiency (n = 3). (D) TRP loading efficiency analysis (1 µM TRP at varying volumes onto 1 mL MMS) (n = 3). (E) Optical microscopy images demonstrating color changes during MMS@TRP fabrication; TEM images illustrating microsphere morphology and surface modifications, where blue arrows indicate the MPN coating and red arrows mark surface‐loaded TRP. (F) TEM elemental mapping of MMS@TRP, highlighting distributions of phosphorus (P), sulfur (S), and strontium (Sr). (G) Fluorescence colocalization of Cy5‐tFNAs and FAM‐miR‐21 inhibitor on MMS@TRP microspheres. (H) FTIR spectra of MS, MMS, and MMS@TRP; Light blue bars represent MPN‐derived metal‐O vibrations (<800 cm^−1^), pink bars indicate MPN‐derived C─O vibrations (1200–1300 cm^−1^), and orange bars highlight TRP‐derived phosphorus‐related peaks (1220–1240 cm^−1^). (I) XPS spectra of MS, MMS, and MMS@TRP, with highlighted peaks at 130–140 eV. (J) Molecular docking and colocalization analyses of TA, DNA, and DNase I. (K, L) Molecular docking illustrations showing interactions between TA and DNase I. (M) PAGE analysis of TA concentration‐dependent inhibition of tFNA degradation in 10% FBS at 24 h, and protective effects of 40 µg/mL TA on TRP stability in 10% FBS over time. Quantitative data are presented as mean ± SD. Error bars represent SD.

First, GelMA synthesis was achieved by grafting gelatin with methacrylic anhydride (MA), confirmed by nuclear magnetic resonance (NMR) spectroscopy (Figure S10A). Subsequently, GelMA microspheres (∼150 µm diameter) were fabricated using microfluidic technology (Figure 6B). Lyophilized and rehydrated GelMA MS were then surface‐modified with TA/Sr^2+^‐based MPNs under alkaline conditions (pH 8.0) to yield MPN‐functionalized MS (MMS). To demonstrate the versatility of MPN‐mediated surface modification, MS prepared from various materials [methacrylated hyaluronic acid (HAMA), GelMA, chondroitin sulfate (SA), chitosan] were successfully functionalized using multiple polyphenols [TA, tea polyphenols (TP), or epigallocatechin gallate (EGCG)], evident from distinct colorimetric changes due to polyphenol‐metal ion chelation (Figure S10B). Subsequently, TRP‐loaded MMS microgels (MMS@TRP) were prepared by incubating TRP with MMS (Figure 6A).

Based on evaluations of DPPH radical‐scavenging activity and TRP loading efficiency, a concentration of 0.5 mg/mL TA/Sr^2+^ was identified as optimal for MMS preparation (Figure 6C; Figure S10C). TRP loading assays indicated that MMS@TRP microgels achieved a maximum TRP loading of 630 nm (Figure 6D). Under light microscopy, MMS@TRP exhibited a pale green appearance similar to MMS following TRP loading (Figure 6E). Scanning electron microscopy (SEM) revealed that lyophilized GelMA MS possessed smooth porous surfaces; after MPN modification, surfaces of MMS showed a distinct rough coating layer, with uniformly distributed TRP particles observed on MMS@TRP surfaces (Figure 6E). SEM elemental mapping confirmed successful MPN modification by detecting strontium (Sr), while phosphorus (P, nucleic acids) and sulfur (S, pPB) signals verified TRP loading (Figure 6F). Fluorescence colocalization analysis using confocal laser scanning microscopy (CLSM) with FAM‐miR‐21 inhibitor and Cy5‐tFNAs further supported successful TRP incorporation (Figure 6G). Fourier‐transform infrared spectroscopy (FTIR) identified characteristic peak shifts following MPN coating (C─O vibrations: 1200–1300 cm^−1^; metal‐O vibrations: <800 cm^−1^) and distinctive phosphorus‐related peaks (1240–1220 cm^−1^) due to TRP loading (Figure 6H). X‐ray photoelectron spectroscopy (XPS) analysis revealed new Sr3d peaks (∼132 eV), indicating successful MPN functionalization. Additionally, sulfur peaks (S2p, 163–165 eV) associated with pPB and phosphate peaks (P2p, 130–140 eV) resulting from TRP merged with the Sr3d peaks to form composite signals, further confirming the TRP loading (Figure 6I). Collectively, these results confirmed successful preparation of the MMS@TRP microgel delivery system, achieved by MPN‐mediated surface modification of GelMA microspheres and subsequent TRP loading. Furthermore, in vitro degradation analysis showed progressive degradation of MMS@TRP over time, with degradation exceeding 80% by 4 weeks in the collagenase II solution and by 6 weeks in the 10% FBS‐containing medium (Figure S11A,B).

Polyphenols have demonstrated the capability to inhibit various enzymatic degradation processes in biological systems [71, 72]. To investigate whether TA could inhibit DNase activity and consequently protect TRP from degradation, molecular docking between TA and DNase I was performed. Co‐localization analysis indicated that TA occupied the DNA‐binding domain of DNase I (Figure 6J). Furthermore, TA showed stable interactions with multiple domains of DNase I, exhibiting the strongest binding affinity (interaction energy: ‐9.3 kcal/mol) at the DNA‐binding domain. The calculated inhibition constant (Ki) was 149.791 nm at 298 K, with both lower‐bound (RMSD l.b.) and upper‐bound (RMSD u.b.) root‐mean‐square deviation values near zero. Binding sites for TA included the critical catalytic residue His252 (essential for DNA hydrolysis) and DNA‐binding residues Tyr134 and Arg41 (Figure 6K,L). To experimentally validate the protective effect of TA against DNase‐mediated degradation, 10% fetal bovine serum (FBS) was utilized to simulate DNase‐rich physiological conditions. PAGE analysis demonstrated a concentration‐dependent inhibitory effect of TA on tFNA degradation in FBS (Figure 6M). Without TA, TRP degradation was nearly complete within 24 h. However, addition of TA at a cell‐compatible concentration (40 µg/mL) significantly reduced TRP degradation, with partial TRP stability maintained even at 48 h (Figure 6M). CLSM fluorescence colocalization analysis of MMS@TRP further demonstrated that, under FBS‐rich conditions, TRP remained immobilized on TA‐functionalized microspheres (MMS@TRP) for over 5 days. In addition, the protective effect of TA on the miR‐21 inhibitor was further evaluated by molecular docking simulation and PAGE analysis. TA exhibited strong binding affinity toward RNase A (interaction energy: ‐8.4 kcal/mol), targeting the RNA‐binding region and interacting with key catalytic residues, including His12, His119, and Lys41 (Figure S12A). Consistently, PAGE analysis demonstrated that TA significantly protected the miR‐21 inhibitor from serum‐mediated degradation, thereby preserving RNA integrity under enzymatic degradation conditions (Figure S12B). Collectively, these findings indicate that GelMA MS functionalized with TA/Sr^2^^+^‐based MPNs establishes an effective protective barrier against nuclease‐mediated degradation, thereby prolonging the stability of both TRP and the therapeutic miR‐21 inhibitor and improving the delivery efficiency and therapeutic potential.

### 2.6 TRP and TA Release From MMS@TRP

Prior to assessing release kinetics, the biocompatibility of the microgel system was evaluated. CCK‐8 assays indicated that NPCs exposed to MS, MMS, or MMS@TRP exhibited comparable growth profiles to untreated controls, confirming minimal cytotoxicity (Figure S13A). Live/dead staining further confirmed the cell viability following microgel treatment (Figure S13B).

The pH‐responsive properties of MPNs facilitate synchronized and stimulus‐triggered release of TRP and TA in the acidic microenvironment characteristic of IVDD. Accordingly, release profiles were evaluated under physiological (pH 7.4) and acidic (pH 6.2) conditions. After 24 h, approximately 32% of TRP (quantified via Cy3 fluorescence using a BioTek Synergy H1 reader) was released at physiological pH, whereas nearly 65% was released at acidic pH. After three days, cumulative TRP release reached approximately 85% at pH 6.2 (Figure 7A). In contrast, TA release (quantified using UV–vis spectrophotometry at 280 nm) displayed a more sustained profile. Approximately 25% of TA was released at physiological pH over three days, while acidic conditions promoted greater than 55% release. Notably, TA continued releasing steadily for up to 14 days at acidic pH (Figure 7B).

**FIGURE 7:**
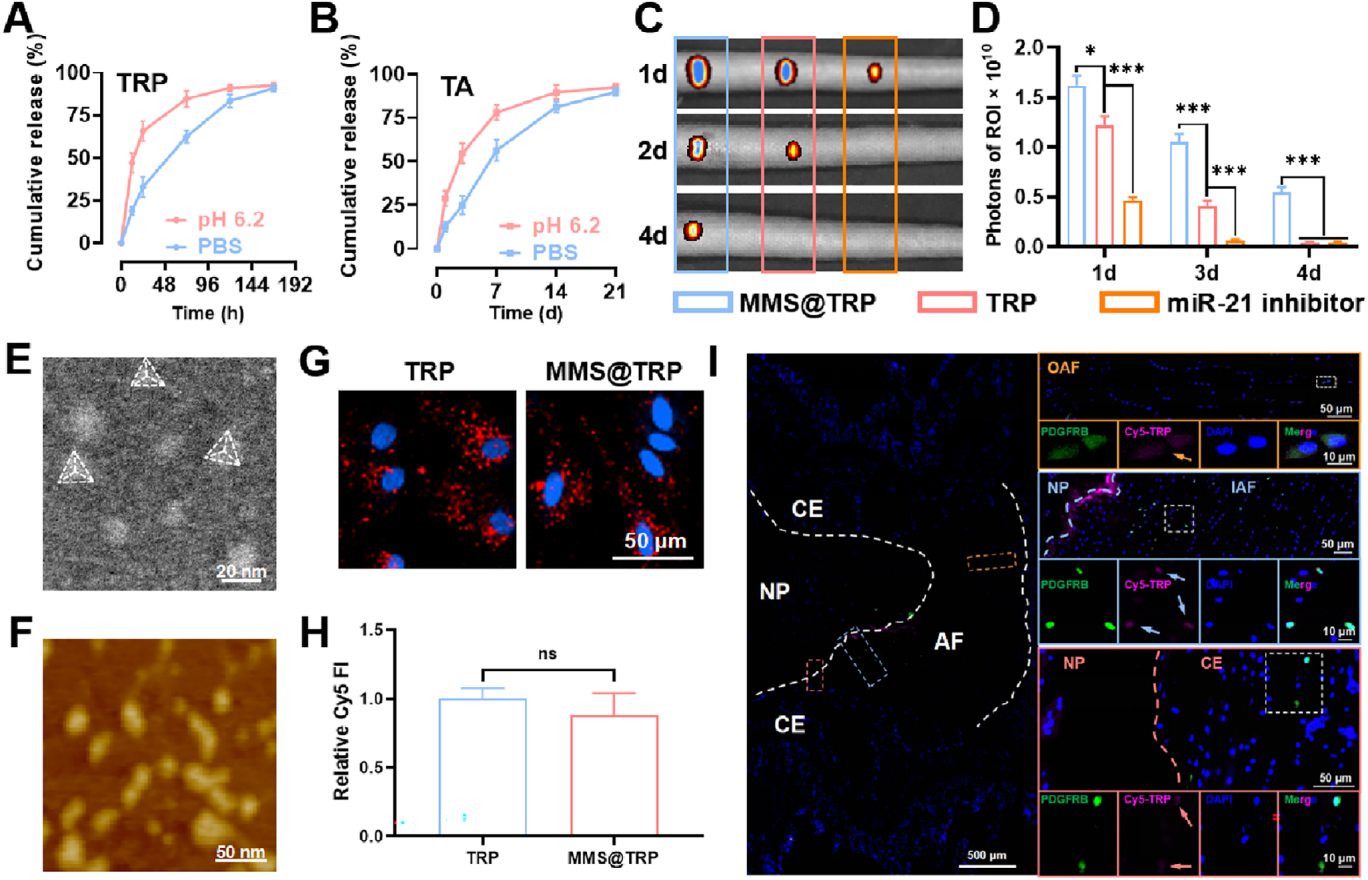
TRP and TA release from MMS@TRP and targeted TRP delivery. (A) TRP release kinetics under physiological (pH 7.4) and acidic (pH 6.2) conditions (n = 3). (B) TA release kinetics under physiological (pH 7.4) and acidic (pH 6.2) conditions (n = 3). (C, D) IVIS Spectrum imaging analysis demonstrating the in vivo retention of the Cy3‐miR‐21 inhibitor over time (n = 3). (E) TEM characterization of TRP morphology released from MMS@TRP. (F) AFM images depicting the nanostructure of TRP released from MMS@TRP. (G, H) Fluorescence‐based comparison of cellular uptake efficiency in pericytes between free Cy5‐TRP and Cy5‐TRP released from MMS@TRP (n = 3). (I) Fluorescence colocalization analysis demonstrating targeted uptake of released Cy5‐TRP by PDGFRB+ pericytes in the outer AF, inner AF, and CE regions following MMS@TRP injection. Quantitative data are presented as mean ± SD. Error bars represent SD. Statistics: two‐way ANOVA with Tukey's multiple‐comparisons test was used for panel (D); unpaired two‐tailed Student's t‐test for panel (H). Significance: ^*^ *p* < 0.05, ^***^ *p* < 0.001; ns, not significant.

The in vivo retention of the miR‐21 inhibitor was assessed by injecting Cy3‐miR‐21 inhibitor‐loaded MMS@TRP microgels into IVD tissues, with subsequent monitoring via an IVIS Spectrum imaging system. As shown in Figure 7C,D, strong Cy3 fluorescence signals were maintained within the disc space of the MMS@TRP‐treated group for more than four days, significantly surpassing those observed with free miR‐21 inhibitor or free TRP alone. Furthermore, fluorescence imaging of major organs (heart, lung, liver, spleen, kidney, and brain) at 1, 2, and 4 days after injection of MMS@TRP microgels showed no apparent accumulation of fluorescence signals, suggesting limited systemic distribution and a low potential for off‐target effects following local administration of MMS@TRP (Figure S14).

These findings highlight the microgel platform's enhanced retention capability, supporting sustained in vivo therapeutic delivery of TRP. The prolonged in vivo retention observed can be attributed to two primary factors: (i) sustained release provided by the MMS@TRP microgel, consistent with previous reports on similar microgel‐based delivery platforms, and (ii) protective effects of TA, which inhibits enzymatic degradation of TRP, as demonstrated above [63, 73]. Collectively, both in vitro release kinetics and in vivo retention analyses confirm the responsive‐release characteristics of MMS@TRP, facilitating initial controlled TRP release and sustained TA delivery in the acidic IVDD microenvironment. Thus, the early release of TRP to inhibit pathological fibrosis in pericytes, combined with long‐term protective effects of TA on NPCs, represents an optimal therapeutic strategy for IVDD. Nevertheless, mechanical loading has been reported to influence the deformation and cargo release of hydrogel‐based delivery systems, particularly in mechanically responsive platforms [74]. Although pH‐responsive destabilization of the TA/Sr^2^^+^ coordination network mediates TRP release from MMS@TRP, the potential effects of repetitive mechanical loading on microgel stability and release kinetics remain to be determined. Therefore, further studies evaluating the mechanical stability and release behavior of MMS@TRP under physiologically relevant IVD mechanical loading conditions are warranted.

To investigate whether TRP release from MMS@TRP affects its nanostructural integrity and internalization efficiency in pericytes, the morphology of released TRP and its cellular uptake efficiency were evaluated. TEM and AFM analyses revealed that TRP released from MMS@TRP maintained its well‐defined nanostructure (Figure 7E,F). Cellular uptake assays further demonstrated that released TRP exhibited internalization efficiency in pericytes comparable to free TRP (Figure 7G,H). These results confirm that the MPN‐functionalized microgel system does not negatively impact the structural integrity or cellular uptake capability of TRP. To further evaluate targeted in vivo delivery, MMS@TRP (Cy5‐TRP) or MMS@TR (Cy5‐TR) were injected into an IVDD rat model. Fluorescence imaging indicated prominent localization of Cy5‐TRP within PDGFRB+ pericytes across various IVD regions, including the CE, IAF, and OAF (Figure 7I). In contrast, no significant enrichment within pericytes was observed with MMS@TR treatment (Figure S15). These results confirm the effective tissue penetration and targeted pericyte‐specific delivery of TRP by the MMS@TRP platform.

### 2.7 MMS@TRP Inhibits Pro‐Fibrotic Behaviors of Pericytes Induced by dNPCs

In this study, TGFβ1 secreted by dNPCs was identified as the primary cytokine responsible for inducing pericyte proliferation, migration, and transition to a pro‐fibrotic phenotype. However, dNPCs are not the only source contributing to elevated TGFβ1 levels within the IVDD microenvironment; other cells, including macrophages and AF cells, also exhibit increased TGFβ1 expression during IVDD [35, 75]. Moreover, pericytes that transition into myofibroblasts can further amplify TGFβ1 signaling, perpetuating fibrosis. Additionally, inflammatory mediators such as MMPs, IL‐1β, and ROS within the degenerative microenvironment activate the TGF‐β pathway and enhance pericyte fibrotic transition [35, 76]. Therefore, directly targeting pericytes represents a promising therapeutic approach. The principal aim of this study was to develop the MMS@TRP microgel delivery system for targeted delivery of miR‐21 inhibitors to pericytes, thus suppressing their proliferation, migration, and fibrotic transformation, and ultimately alleviating pathological fibrosis in IVDD (Figure 8A).

**FIGURE 8:**
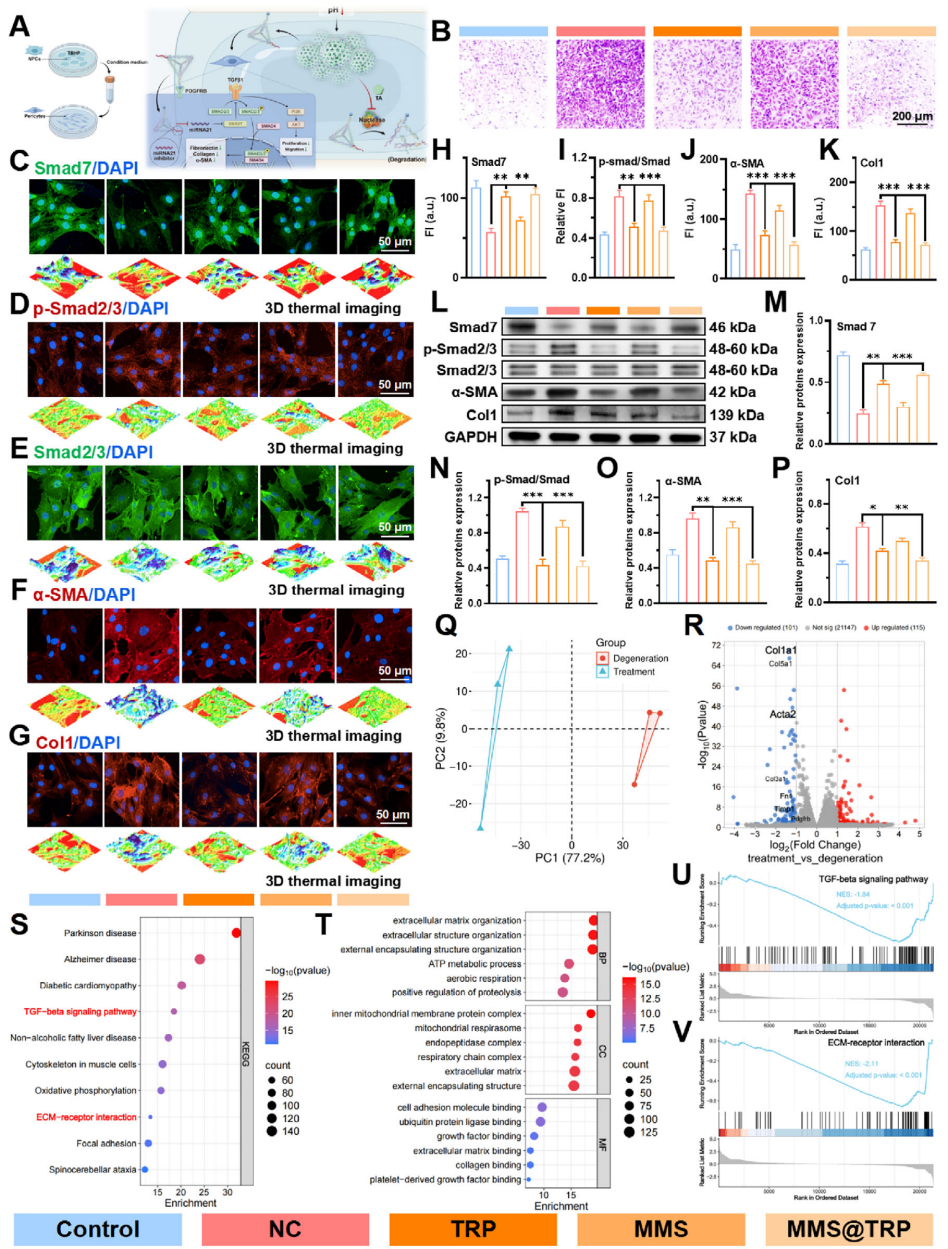
MMS@TRP inhibits pericyte activation and pro‐fibrotic phenotypic transition. (A) Schematic illustrating the therapeutic mechanism of MMS@TRP in pericytes. (B) Transwell migration assay evaluating pericyte migration inhibition by TRP, MMS, or MMS@TRP. (C‐K) Immunofluorescence staining and quantitative analyses of Smad7, p‐Smad2/3, Smad2/3, α‐SMA, and Col1 expression in treated pericytes. (L‐P) Western blot analyses and quantification of Smad7, p‐Smad2/3, Smad2/3, α‐SMA, and Col1 expression following treatments. (Q) PCA illustrating transcriptional profile alterations induced by MMS@TRP treatment. (R) Volcano plot demonstrating expression changes in genes related to pericyte‐to‐myofibroblast transition after MMS@TRP exposure. (S) KEGG enrichment analysis identifying significant signaling pathways among DEGs. (T) GO enrichment analysis highlighting DEGs associated with BP, CC, and MF. (U, V) GSEA demonstrating inhibition of TGF‐β signaling and ECM‐receptor interaction in pericytes treated with MMS@TRP. Quantitative data are presented as mean ± SD. Error bars represent SD. Statistics: one‐way ANOVA with Tukey's multiple‐comparisons test was used for panels (H‐K and M‐P). Significance: ^*^ *p* < 0.05, ^**^ *p* < 0.01, ^***^ *p* < 0.001.

To evaluate the therapeutic efficacy of MMS@TRP on pericytes, in vitro experiments using dNPC‐treated pericytes were performed (Figure 8A). CCK‐8 assays indicated significant inhibition of pericyte proliferation by both TRP and MMS@TRP treatments (Figure S16A). Migration assays further demonstrated that both TRP and MMS@TRP effectively reduced pericyte migration (Figure 8B). Immunofluorescence staining revealed that compared to controls, treatment with TRP or MMS@TRP markedly elevated Smad7 expression in pericytes (Figure 8C,H). Correspondingly, phosphorylation levels of Smad2/3 significantly decreased in both treatment groups (Figure 8D,E,I). Furthermore, pericyte transition to myofibroblasts was substantially inhibited, evident from reduced expression of α‐SMA and Col1 (Figure 8F,G,J,K). Western blot analysis corroborated these findings (Figure 8L–P). Collectively, these results demonstrate that MMS@TRP effectively suppresses pericyte proliferation, migration, and pro‐fibrotic transition induced by dNPCs, mediated by miR‐21 inhibitor delivery, subsequent Smad7 upregulation, and inhibition of TGFβ signaling. Additionally, MMS alone exhibited mild inhibitory effects, likely attributable to TA's ROS‐scavenging and anti‐inflammatory properties within the microgel.

To further elucidate the molecular mechanisms underpinning MMS@TRP treatment, RNA‐seq analysis was conducted. Principal component analysis (PCA) revealed distinct alterations in gene expression profiles of dNPC‐treated pericytes following MMS@TRP exposure (Figure 8Q). Volcano plot analysis showed significant downregulation of myofibroblast markers and fibrosis‐related genes, including α‐SMA, Col1, and Fn1, in the MMS@TRP group (Figure 8R). KEGG enrichment analysis indicated that DEGs were primarily associated with the TGF‐β signaling pathway, cytoskeleton in muscle cells, ECM‐receptor interaction, and focal adhesion (Figure 8S). GO enrichment analysis further confirmed that DEGs were predominantly involved in BP related to ECM organization and extracellular structure formation, CC associated with ECM, and MF involving ECM, cell adhesion molecules, and collagen binding (Figure 8T). In addition, GSEA demonstrated significant inhibition of TGF‐β signaling, ECM‐receptor interaction, and the PI3K‐Akt signaling pathway upon MMS@TRP treatment (Figure 8U,V; Figure S16B). These data collectively confirm that MMS@TRP suppresses pericyte proliferation, migration, and fibrotic phenotype transition by blocking canonical TGF‐β/Smad and non‐canonical PI3K‐Akt signaling pathways (Figure 8A). In conclusion, MMS@TRP provides an effective therapeutic strategy for mitigating pericyte‐driven pathological fibrosis in IVDD.

### 2.8 MMS@TRP Microgel Protects NPCs Through ROS Scavenging, Mitochondrial Protection, and Anti‐Inflammatory

NPCs are critical cells required to maintain IVD health; thus, protecting NPCs is essential for effective IVDD treatment. Previous studies have demonstrated that TA can protect NPCs through ROS scavenging, mitochondrial protection, and anti‐inflammatory actions [54, 69, 70]. Moreover, TA possesses abundant active phenolic groups capable of interacting with biomaterials, making it a widely used crosslinker to enhance the performance of biomaterial‐based delivery systems [77, 78]. In this study, TA was incorporated into the MMS@TRP microgel both as a protective mediator for TRP delivery and as a bioactive component to safeguard NPC function upon its release. We thus evaluated the protective effects of MMS@TRP on NPCs. CCK‐8 assays and live/dead staining confirmed that MMS and MMS@TRP significantly enhanced NPC viability and proliferation under oxidative stress conditions induced by TBHP (Figure S17A,B). In the inflammatory microenvironment of IVDD, elevated ROS disrupt mitochondrial function by inducing mitochondrial permeability transition pore opening, reducing mitochondrial membrane potential (ΔΨm), and causing mitochondrial swelling, cristae loss, and respiratory chain dysfunction [79, 80]. These alterations further elevate intracellular ROS levels, activate inflammatory pathways, and ultimately impair NPC function [81]. To confirm the antioxidant properties of MMS@TRP, we assessed extracellular and intracellular ROS scavenging capabilities. DPPH, PTIO, and ABTS scavenging assays demonstrated that MMS and MMS@TRP exhibited potent extracellular ROS‐scavenging efficiency (Figure 9B–D). Additionally, the DCFH‐DA probe assay revealed that MMS and MMS@TRP markedly reduced total cytoplasmic ROS in dNPCs (Figure 9E,H). In terms of mitochondrial protection, both MMS and MMS@TRP effectively preserved ΔΨm and suppressed mitochondrial ROS (mtROS) production in dNPCs (Figure 9F,G,I,J).

**FIGURE 9:**
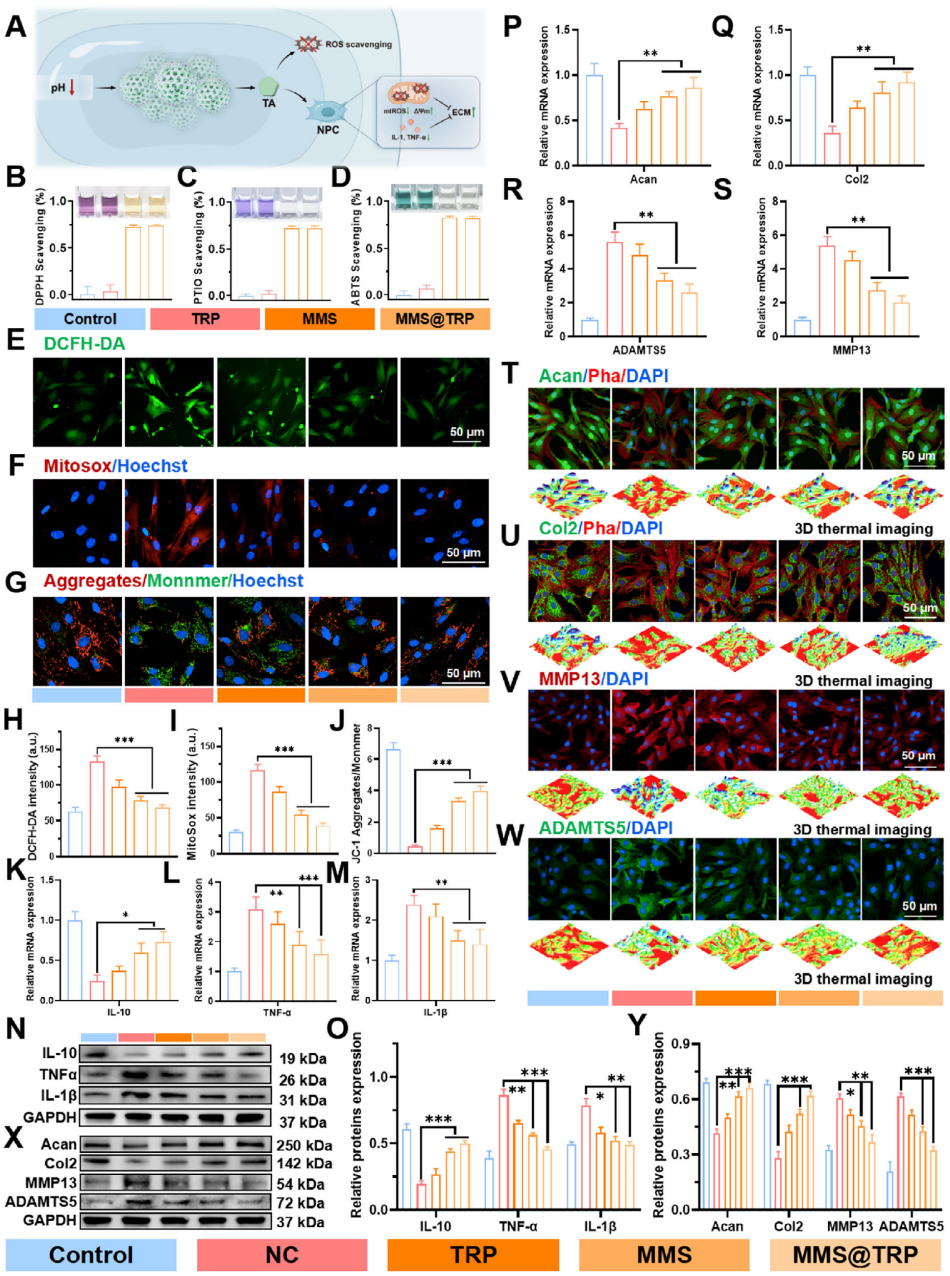
MMS@TRP protects NPCs through ROS scavenging, mitochondrial protection, and anti‐inflammatory effects. (A) Schematic illustration depicting NPC protection mechanisms mediated by MMS@TRP. (B‐D) DPPH, PTIO, and ABTS assays showing extracellular ROS‐scavenging activities of TRP, MMS, and MMS@TRP (n = 3). (E) DCFH‐DA assay detecting total intracellular ROS scavenging in NPCs treated with TRP, MMS, or MMS@TRP. (F) MitoSOX staining illustrating mitochondrial ROS (mtROS) levels in NPCs. (G) JC‐1 staining indicating ΔΨm preservation in NPCs. (H) Quantification of total intracellular ROS levels (n = 3). (I) Quantitative analysis of mtROS production (n = 3). (J) Quantitative analysis of ΔΨm (n = 3). (K‐M) RT‐qPCR quantifying expression levels of inflammatory cytokines IL‐10, TNF‐α, and IL‐1β in NPCs (n = 3). (N,O) Western blot analysis quantifying protein levels of inflammatory cytokines IL‐10, TNF‐α, and IL‐1β in NPCs (n = 3). (P,Q) RT‐qPCR analysis of ECM anabolic markers Acan and Col2 in NPCs. (R,S) RT‐qPCR analysis of ECM catabolic markers MMP13 and ADAMTS5 in NPCs. (T‐W) Immunofluorescence staining of ECM anabolic markers (Acan, Col2) and ECM catabolic markers (MMP13, ADAMTS5) in NPCs. (X,Y) Western blot analysis quantifying ECM anabolic markers (Acan, Col2) and catabolic markers (MMP13, ADAMTS5) expression in NPCs (n = 3). Data are presented as mean ± SD. Error bars represent SD. Statistics: one‐way ANOVA with Tukey's multiple‐comparisons test was used. Statistical significance is indicated as ns (no significance), ^*^ *p* < 0.05, ^**^ *p* < 0.01, and ^***^ *p* < 0.001.

Mitochondrial dysfunction and inflammatory pathway activation exacerbate cellular senescence, apoptosis, impaired NPC function, and ECM synthesis disruption [82]. Elevated ROS levels and pro‐inflammatory cytokines also stimulate ECM‐degrading enzymes (e.g., MMP13 and ADAMTS5), accelerating ECM degradation and further disrupting ECM homeostasis [53]. We therefore evaluated the anti‐inflammatory effects of MMS@TRP by quantifying inflammatory cytokine expression. RT‐qPCR analysis indicated that MMS@TRP significantly decreased IL‐1β and TNF‐α levels while elevating IL‐10 expression in dNPCs (Figure 9K–M). Western blotting and immunofluorescence staining corroborated these findings (Figure 8N,O; Figure S17C–F). Additionally, we investigated the impact of MMS@TRP on ECM metabolism in NPCs. Results from RT‐qPCR, immunofluorescence, and western blot analyses consistently showed that MMS@TRP significantly enhanced expression of anabolic ECM components (Col2 and Acan) while inhibiting ECM‐degrading enzymes (MMP13 and ADAMTS5) (Figure 9P–Y; Figure S17G,H). Additionally, TRP alone exhibited moderate protective effects on NPCs by reducing intracellular ROS, suppressing inflammation, and promoting ECM synthesis. These effects can be attributed to tFNAs, which protect mitochondrial function by inhibiting Drp1‐mediated mitochondrial fission, enhancing mitochondrial oxidative phosphorylation, regulating endoplasmic reticulum stress, and increasing antioxidant enzyme expression (SOD, GSH‐Px) [83, 84]. Moreover, miR‐21 inhibitors regulate ECM metabolism in NPCs via inhibition of the MEK/ERK signaling pathway [54]. However, TRP alone lacks direct ROS‐scavenging capabilities, is unable to eliminate environmental ROS, and exhibits lower efficacy compared to MMS or MMS@TRP.

Collectively, these results indicate that MMS@TRP effectively protects NPCs through sustained TA release, thereby scavenging ROS, preserving mitochondrial integrity, suppressing inflammation, and regulating ECM metabolism (Figure 9A). This highlights the distinct advantages and necessity of integrating TA within the MMS@TRP microgel delivery platform.

In addition, macrophages are key immune cells involved in maintaining the inflammatory microenvironment of IVDD and also exhibited TRP uptake in vitro (Figure S9). Therefore, the effects of the MMS@TRP platform on macrophages were further evaluated. Immunofluorescence analysis showed that TRP, MMS, and MMS@TRP decreased TNF‐α expression and increased IL‐10 expression to varying degrees, with MMS@TRP exhibiting the strongest effects (Figure S18). The anti‐inflammatory effects of TRP and MMS on macrophages are likely associated with the immunomodulatory properties of tFNAs and TA, respectively, which have been shown to regulate macrophage inflammatory responses and suppress pro‐inflammatory cytokine expression through inflammation‐related signaling pathways [85, 86]. By integrating these effects, MMS@TRP effectively regulated inflammatory cytokine expression in macrophages, further supporting its multifunctional therapeutic potential for ameliorating the inflammatory microenvironment in IVDD.

### 2.9 In Vivo Evaluation of MMS@TRP Microgel Treating IVDD

Considering the promising therapeutic outcomes of MMS@TRP observed in vitro, its efficacy was further evaluated in vivo using a rat IVDD model. After inducing disc degeneration via disc puncture, various treatments were administered intradiscally. Radiographic and histological analyses were conducted at 4 and 8 weeks post‐injection to assess disc recovery (Figure 10A). The disc height index (DHI) from X‐ray imaging and MRI signal intensity served as quantitative measures of disc integrity.

**FIGURE 10:**
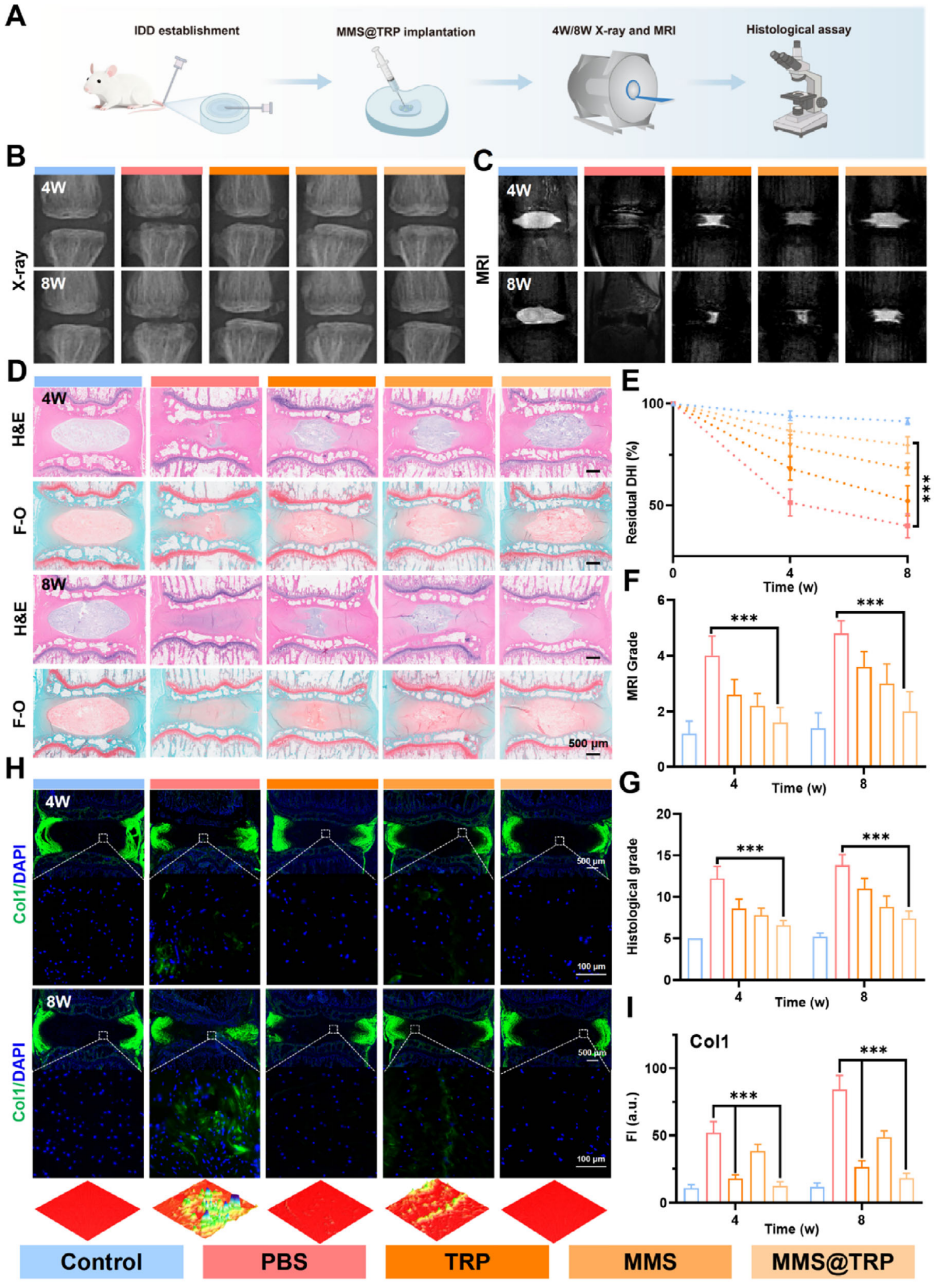
Evaluation of the in vivo therapeutic efficacy of the MMS@TRP system. (A) Experimental design schematic. (B) Representative X‐ray images and (C) MRI images of rat coccygeal discs at 4 and 8 weeks post‐treatment. (D) Representative H&E and Safranin‐O/Fast Green staining across treatment groups at 4 and 8 weeks. (E) Changes in disc height index (DHI) at 4 and 8 weeks (n = 5). (F) MRI grading scores at 4 and 8 weeks (n = 5). (G) Histological grading across treatment groups at 4 and 8 weeks (n = 5). (H, I) Immunofluorescence staining and quantification of fibrotic marker Col1 expression (n = 5). Data are presented as mean ± SD. Error bars represent SD. Statistics: two‐way ANOVA with Tukey's multiple‐comparisons test was used for panels (E,F); one‐way ANOVA with Tukey's multiple‐comparisons test was used for panels(G,I). Significance: ^***^ *p* < 0.001.

X‐ray analyses revealed significant disc height loss in the PBS‐treated group. TRP alone demonstrated limited efficacy, maintaining a significantly lower DHI compared to MMS. However, MMS@TRP most effectively preserved disc height, particularly at 8 weeks (Figure 10B,E). These results highlight the superior capability of the MMS@TRP microgel system in maintaining spinal segment integrity, attributable to ECM supplementation by GelMA MS and the sustained therapeutic action of TA. MRI scans further emphasized MMS@TRP's effectiveness in slowing disc degeneration. Specifically, MMS@TRP‐treated discs showed significantly higher T2‐weighted signal intensities in the NP region at both time points compared to other groups (Figure 10C). Correspondingly, improved MRI‐based Thomson grading scores indicated attenuated IVDD progression (Figure 10F).

Histological staining further supported these findings. H&E staining revealed severe NP atrophy, blurred NP/AF boundaries, and fibrotic replacement in PBS‐treated discs. All treated groups showed varying degrees of NP preservation, with the MMS@TRP group displaying the most intact disc morphology and tissue organization (Figure 10D). Safranin‐O/Fast Green staining demonstrated significant preservation of proteoglycans in MMS@TRP‐treated discs, in contrast to extensive proteoglycan loss and collagen replacement in the PBS group. The MMS group displayed moderate proteoglycan preservation, primarily due to the protective effects of TA on NPCs, as previously shown in vitro (Figure 10D). Histological scoring confirmed the protective effects of MMS@TRP against IVDD progression (Figure 10G). Although Sr^2^^+^ is widely recognized for its osteogenic activity, no abnormal calcification was observed following MMS@TRP or MMS treatment, supporting the feasibility of TA/Sr^2^^+^‐based MPNs for IVDD treatment under the present formulation and treatment conditions [87].

Immunofluorescence assays provided molecular‐level evidence of the anti‐fibrotic effects, ECM preservation, and anti‐inflammatory properties of the MMS@TRP microgel system. Expression of Col1, a fibrotic marker, was significantly reduced by TRP and MMS@TRP treatments compared to the PBS group at both 4 and 8 weeks (Figure 10H,I). Additionally, MMS alone exhibited anti‐fibrotic effects through its protective impact on NPCs, thereby inhibiting their myofibroblast transition (Figure 10H,I). Moreover, TA release from MMS may attenuate pericyte pro‐fibrotic transitions triggered by ROS, MMPs, and IL‐1β in the inflammatory IVDD microenvironment [35, 73]. However, MMS demonstrated inferior anti‐fibrotic efficacy compared to TRP and MMS@TRP.

Evaluation of ECM metabolism showed that MMS@TRP significantly increased expression of anabolic ECM components (Acan and Col2) while decreasing catabolic enzymes (MMP13 and ADAMTS5) (Figure 11A–F,H,I; Figure S19A–D). Consistent with in vitro findings, TRP and MMS treatments also improved ECM metabolism, with MMS outperforming TRP. These results indicate comprehensive restoration of ECM homeostasis through the multifunctional MMS@TRP microgel system. Additionally, immunofluorescence analysis demonstrated significantly reduced TNF‐α expression in both MMS and MMS@TRP groups (Figure 11G,I; Figure S19E), confirming their anti‐inflammatory capacity, mediated primarily by sustained TA release.

**FIGURE 11:**
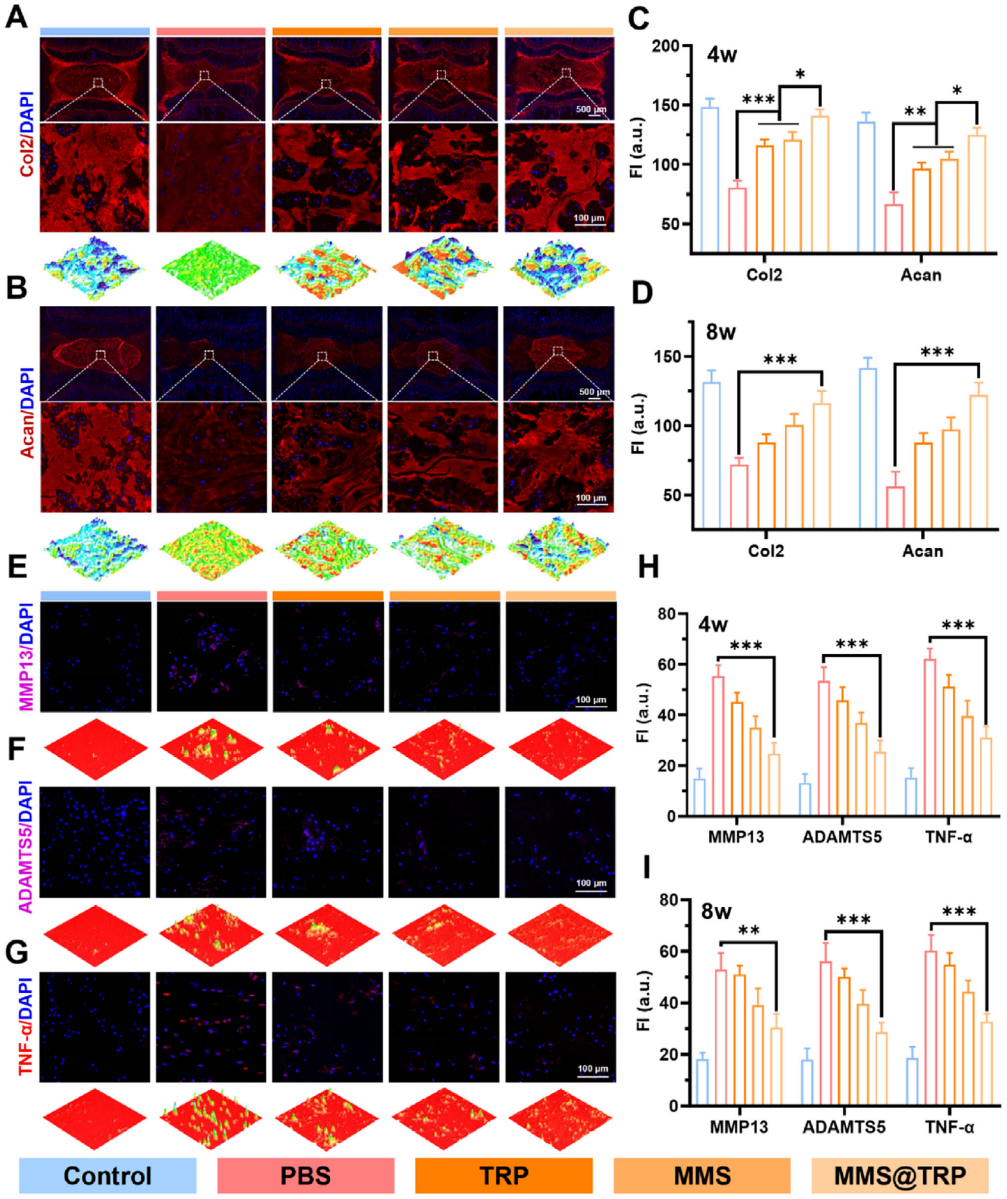
Analysis of the in vivo therapeutic efficacy of MMS@TRP treatment in a rat model of IVDD. (A‐C) Immunofluorescence staining and quantitative analysis of key ECM anabolic components (Acan and Col2) at 4 weeks (n = 5). (D) Quantitative analysis of Acan and Col2 immunofluorescence at 8 weeks (n = 5). (E‐G) Immunofluorescence staining of ECM catabolic enzymes (MMP13 and ADAMTS5) and inflammatory factor (TNF‐α) at 4 weeks. (H,I) Quantitative immunofluorescence analysis of indicated markers across treatment groups at 8 weeks (n = 5). Data are presented as mean ± SD. Error bars represent SD. Statistics: one‐way ANOVA with Tukey's multiple‐comparisons test was used. Statistical significance is indicated as ^*^ *p* < 0.05, ^**^ *p* < 0.01, and ^***^ *p* < 0.001.

To further assess the therapeutic advantage of PDGFRB‐mediated pericyte targeting, histological and immunohistochemical comparisons between MMS@TR and MMS@TRP were performed at 8 weeks after treatment. H&E and Safranin‐O/Fast Green staining showed relatively better preservation of IVD structure and proteoglycan content in the MMS@TRP group, although no significant difference in histological scores was observed (Figure S20A,B). Importantly, MMS@TRP significantly reduced Col1 expression and increased Col2 expression compared with MMS@TR, supporting the contribution of PDGFRB‐mediated pericyte targeting to the enhanced anti‐fibrotic efficacy of MMS@TRP (Figure S20C–F).

Given the biomechanically demanding and load‐bearing nature of the intervertebral disc, evaluating the restoration of its biomechanical function is important for assessing therapeutic efficacy. Therefore, axial compression testing was performed on ex vivo vertebra–disc–vertebra motion segments at 8 weeks after treatment. The stress–strain curves showed a marked alteration in the compressive response of IVDD motion segments, with substantially higher stress at corresponding strain levels than the normal group, indicating reduced deformation and compressive buffering capacities of the degenerated discs. In contrast, MMS@TRP treatment restored the stress–strain response toward that of the normal group, supporting effective preservation of disc biomechanical function (Figure S21A,B).

Furthermore, the systemic biosafety of MMS@TRP microgels was evaluated by hemolysis testing and histological examination of major organs. MMS@TRP exhibited favorable systemic biosafety, with hemolysis rates below 5% and no obvious histopathological abnormalities in major organs, including the heart, liver, spleen, lungs, and kidneys, at 8 weeks after treatment (Figure S22A,B). These findings further support the long‐term biocompatibility and systemic safety of MMS@TRP.

Collectively, these in vivo findings demonstrate that MMS@TRP alleviates IVDD progression through coordinated pericyte‐targeted anti‐fibrotic intervention and sustained TA‐mediated protection of NPCs. The observed preservation of disc biomechanical function further supports its therapeutic efficacy. Given its pH‐responsive release characteristics, MMS@TRP represents a promising multifunctional strategy for suppressing pathological fibrosis and preserving intervertebral disc homeostasis. Nevertheless, future studies in large‐animal models, together with comparisons with representative delivery strategies and assessments of pain‐related behavioral outcomes, are needed to further establish its therapeutic advantages and translational potential.

## 3 Conclusion

This study provides the first demonstration that dNPCs drive pathological fibrotic remodeling in IVDD by activating the TGF‐β signaling pathway in pericytes, thereby enhancing their proliferation, migration, and transition into myofibroblasts. Furthermore, a miR‐21 inhibitor was shown to suppress pericyte activation and pro‐fibrotic transition by upregulating Smad7 and subsequently blocking TGF‐β signaling. These findings identify pericytes as novel therapeutic targets for anti‐fibrotic intervention in IVDD.

Building on these insights, we designed a targeted nanocarrier (TRP) for miR‐21 inhibitor delivery, employing the PDGFRB‐specific peptide pPB. TRP demonstrated efficient penetration into the IVD with precise targeting capability, effectively inhibiting pericyte‐driven fibrosis. The microgel system, functionalized with metal‐phenolic networks (MPNs) incorporating active phenolic hydroxyl groups, provides a versatile and efficient approach for nanoparticle loading and environmentally responsive, controlled release within acidic microenvironments. Additionally, the incorporation of TA‐based MPNs enhances microgel versatility by inhibiting nuclease activity, providing a protective environment for nucleic acid nanocarriers and the therapeutic miR‐21 inhibitor. In this study, TA within the MMS@TRP system functions both as a crosslinker and protective agent for TRP delivery and as an active therapeutic molecule to safeguard NPCs. In vitro and in vivo experiments collectively demonstrated that this multifunctional microgel system effectively inhibits pathological fibrosis by targeting pericytes early with TRP and provides sustained TA release to protect NPCs. Therefore, the MMS@TRP microgel platform presents a promising and effective therapeutic approach for combating IVDD.

## 4 Experimental Section

### 4.1 Materials

Gelatin, methacrylic anhydride (MA), chondroitin sulfate (CS), chitosan, strontium chloride hexahydrate (SrCl_2_·6H_2_O), tannic acid (TA), tea polyphenols (TP), and epigallocatechin gallate (EGCG) were procured from Macklin (Shanghai, China). The designed single‐stranded oligonucleotides, pBP, and all primer sequences in our study were synthesized and purchased from Sangon Biotechnology (Shanghai, China). Gene Pharma (Shanghai, China) synthesized the miRNA and miRNA inhibitor oligonucleotides. Cell culture consumables, including media, trypsin, PBS, enzyme‐free water, plates, and dishes, were purchased from Corning (Shanghai, China), while Abcam (Shanghai, China) and Abclonal (Wuhan, China) provided the antibodies. Furthermore, live/dead staining kits, phalloidin, Hoechst, and DAPI were purchased from Solarbio (Beijing, China).

### 4.2 scRNA‐seq Analysis

The scRNA‐seq dataset was obtained from the National Omics Data Encyclopedia (accession number: OEP003834) [24]. Data integration, dimensionality reduction, and clustering were performed using the Python package Scanpy (v1.9.1) [24]. Batch effects across datasets were corrected using the BBKNN function. Uniform manifold approximation and projection (UMAP) was employed for dimensionality reduction, and cell clusters were identified using the Leiden algorithm. Differentially expressed genes (DEGs) among clusters were identified using the sc.tl.rank_genes_groups function in Scanpy. Cell clusters were annotated based on previously reported signature genes. Subsequent analyses focused specifically on pericytes, including visualization of cell proportions, volcano plots of DEGs, Kyoto Encyclopedia of Genes and Genomes (KEGG) enrichment analysis, and gene set enrichment analysis (GSEA).

### 4.3 Detection of Pericytes and Fibrosis Assessment in IVD Tissue

This study was approved by the Ethics Committees of West China Hospital, Sichuan University (Approval No. 20240919005) and the Affiliated Hospital of Southwest Medical University (Approval No. KY2025394). Written informed consent was obtained from all participants permitting collection and scientific use of their tissue samples. Intervertebral disc (IVD) tissues were collected from six patients (aged 34–62 years) undergoing lumbar fusion surgery. Patients had no history of cardiovascular, cerebrovascular, oncological, infectious, immune, endocrine diseases, or organ dysfunction. Preoperative MRI scans were used to classify IVD degeneration based on the Pfirrmann grading system: three patients had mild degeneration (grade II), and three had severe degeneration (grades IV or V). Samples of nucleus pulposus (NP), cartilaginous endplate (CEP), and annulus fibrosus (AF) tissues were collected for analysis. Animal experiments were conducted according to the guidelines of the International Council for Laboratory Animal Science (ICLAS). A rat IVDD model was established using a percutaneous needle puncture and aspiration technique on specific caudal discs, as previously described [88]. Degenerated disc tissues were harvested four weeks post‐procedure for subsequent analysis. Immunofluorescence staining was performed on both human and rat degenerated IVD tissues to detect pericyte markers (Rgs5 and PDGFRB) in NP, CEP, and AF regions. Immunohistochemical staining for Col1 was conducted on human NP tissues, while immunofluorescence staining for Col1 was performed on tissues from the rat model.

### 4.4 Pericyte Transplantation

Primary rat pericytes were transplanted into degenerated discs. Specifically, 20 µL of primary pericytes (5 × 10^6^ cells/mL in PBS) were injected into the IVD one week after model establishment. The control group received the same volume of PBS. Four weeks post‐transplantation, tissues were harvested for analysis. Structural changes and proteoglycan content were evaluated through histological analyses (H&E and Safranin O‐Fast Green staining). Fibrosis (Col1) and pericyte markers (PDGFRB and Rgs5) were assessed using immunofluorescence staining (n = 3).

### 4.5 Mechanistic Analysis of Pericyte Proliferation, Migration, and Fibrotic Phenotype Transition in IVDD

To simulate the IVDD microenvironment in vitro, pericytes were co‐cultured with conditioned medium derived from tert‐butyl hydroperoxide (TBHP)‐induced degenerated nucleus pulposus cells (dNPCs). NPCs were isolated from four‐week‐old Sprague‐Dawley rats as previously described [88]. NPCs at passages 3–6 were used in all experiments. For conditioned medium preparation, NPCs at 70% confluence were treated with 50 µm TBHP for 24 h. The medium was then replaced, and cells were incubated for an additional 48 h. Supernatants were collected and centrifuged at 6000 × g for 10 min at 4°C to remove impurities. The supernatants were subsequently concentrated to one‐fiftieth of the original volume using ultrafiltration tubes (3 kDa cutoff) at 8000 × g for 30 min at 4°C. The concentrated conditioned medium was mixed with pericyte culture medium at a 1:9 volume ratio and applied to pericytes. Pure pericyte culture medium and conditioned medium from non‐degenerated NPCs served as blank and negative controls (NC), respectively. For the TGFβ1 antibody neutralization experiment, pericytes were treated with a conditioned medium containing 100 nM TGFβ1 monoclonal antibody.

Pericyte proliferation was evaluated at 24 and 48 h using the CCK‐8 assay. For the CCK‐8 assay, the supernatant was removed, and cells were washed three times with fresh medium. Cells were subsequently incubated with 100 µL complete medium containing 10% CCK‐8 reagent at 37°C for 2 h. For migration assays, pericytes were seeded in the upper chamber of Transwell inserts, while medium containing dNPC‐derived supernatant was added to the lower chamber as a chemoattractant. Cell migration was assessed after 48 h. Pericyte‐to‐myofibroblast transition was evaluated by immunofluorescence staining for α‐SMA and Col1 after 48 h of dNPC‐conditioned medium treatment. Transcriptomic analyses were performed to investigate the molecular mechanisms underlying dNPC‐induced alterations in pericyte behavior. Total RNA was extracted and assessed for integrity (RIN). Qualified samples were used for library construction and paired‐end sequencing. Differential expression analysis was performed using DESeq2/edgeR (|log2FC| ≥ 1, adjusted p < 0.05), followed by KEGG/GO enrichment analysis and GSEA (n = 3).

To further validate the role of TGFβ signaling in pericyte fibrotic transformation, gain‐ and loss‐of‐function experiments were performed. Pericytes were treated with recombinant TGFβ1 (10 ng/mL) with or without the TGFβ type I receptor inhibitor SB‐431542 (10 µm). In parallel, pericytes were treated with dNPC‐conditioned medium in the presence or absence of SB‐431542 (10 µM). The expression of Smad7, Smad2/3, p‐Smad2/3, α‐SMA, and Col1 was evaluated by western blotting. To further assess the functional contribution of PI3K‐Akt signaling, dNPC‐conditioned medium‐treated pericytes were treated with the PI3K inhibitor LY294002 (25 µm). The expression of Akt, p‐Akt, α‐SMA, and Col1 was evaluated by western blotting, while pericyte proliferation and migration were assessed using CCK‐8 and Transwell assays, respectively (n = 3 or 6).

### 4.6 miR‐21 Inhibitor Transfection and Validation of the miR‐21/Smad7 Axis in Pericytes

To investigate the effects of miR‐21 inhibition in pericytes, cells were first exposed to dNPC‐conditioned medium for 24 h and subsequently transfected with PBS, miR‐21 mimic NC, miR‐21 mimic, miR‐21 inhibitor NC, or miR‐21 inhibitor (200 pmol/mL) for 48 h (n = 3). Transfection was performed using Lipofectamine 3000 (Invitrogen) according to the manufacturer's protocol. Western blotting was performed to determine the expression of Smad7, Smad2/3, p‐Smad2/3, α‐SMA, and Col1.

To further validate the direct interaction between miR‐21 and Smad7, the wild‐type (WT) or mutant (MUT) Smad7 3′‐UTR containing the predicted miR‐21‐binding site was inserted into the pmirGLO luciferase reporter vector. Cells were co‐transfected with the corresponding reporter plasmid (500 ng) and miR‐21 mimic or control mimic (50 nm) using Lipofectamine 3000. After 48 h, dual‐luciferase reporter assays were performed, and relative luciferase activity was calculated by normalizing firefly luciferase activity to Renilla luciferase activity. To further determine whether Smad7 functionally mediates the anti‐fibrotic effects of miR‐21 inhibition, Smad7 was knocked down by transfection with siSmad7 (30 nm) using Lipofectamine 3000 in miR‐21 inhibitor‐treated pericytes. The expression of Smad7, Smad2/3, p‐Smad2/3, α‐SMA, and Col1 was subsequently evaluated by western blotting (n = 3).

### 4.7 Synthesis and Characterization of TRP

Tetrahedral framework nucleic acids (tFNAs) were synthesized using a thermal annealing method according to previously reported protocols [89]. Four single‐stranded DNAs (S1‐S4) (1 µm), including S2‐P containing sticky ends complementary to pPB and S3‐R containing sticky ends complementary to the miR‐21 inhibitor (Table S1), were dissolved in TM buffer containing magnesium ions. The mixture was heated to 95°C and then gradually cooled to 4°C to assemble the tFNAs. Subsequently, the miR‐21 inhibitor and pPB were mixed with tFNAs at equimolar concentrations and incubated at 4°C for 4 h to generate TRP. TR was synthesized using the same procedure without pPB incorporation. Successful synthesis of tFNAs, TR, and TRP was confirmed by polyacrylamide gel electrophoresis (PAGE), high‐performance capillary electrophoresis (HPCE), and UV–visible spectroscopy. Dynamic light scattering (DLS) was used to determine particle size and zeta potential. Transmission electron microscopy (TEM) and atomic force microscopy (AFM) were further employed to characterize particle morphology and structural features.

### 4.8 Cell Uptake of TRP

Pericytes were initially treated with conditioned medium from dNPCs for 24 h. Subsequently, the pericytes were incubated with Cy5‐labeled tFNAs, TR, or TRP for 4 or 8 h. Cellular uptake was assessed by flow cytometry. For intracellular delivery analysis, pericytes were incubated with TRP containing Cy5‐labeled tFNAs and a FAM‐labeled miR‐21 inhibitor for 6 h. Fluorescence colocalization analysis confirmed successful loading of the miR‐21 inhibitor onto TRP and simultaneous delivery into cells. To determine if PDGFRB mediates TRP uptake, pericytes were first treated with dNPC‐conditioned medium and then incubated with 5 µg/mL anti‐PDGFRB antibody at 37°C for 2 h to block the receptor. Cy5‐labeled TRP was subsequently applied for 6 h. In addition, to further evaluate the preferential uptake of TRP by pericytes, Cy5‐labeled TRP was incubated with pericytes, NPCs, and RAW264.7 macrophages under identical conditions for 2 or 6 h. Cellular uptake was evaluated by confocal fluorescence microscopy followed by semiquantitative analysis of intracellular Cy5 fluorescence intensity.

### 4.9 Construction and Characterization of MMS@TRP

Gelatin methacryloyl (GelMA) was synthesized by grafting gelatin with methacrylic anhydride (MA), as previously described, and successful grafting was confirmed by ^1^H NMR analysis [90]. GelMA microgels (MS) were prepared using a microfluidic technique. Briefly, an aqueous phase consisting of 10% (w/v) GelMA solution and 0.5% (w/v) lithium phenyl‐2,4,6‐trimethylbenzoylphosphinate (LAP) in deionized water was prepared. The oil phase comprised paraffin oil containing 4% (w/v) Span80. The flow rates of the aqueous and oil phases were set at 12 and 120 µL/min, respectively, using a syringe pump. The microgels were collected and cross‐linked by exposure to 405 nm light for 5 min. After cross‐linking, the MS were washed three times with deionized water.

TA/Sr^2+^‐based metal‐phenolic networks (MPNs) were used to modify GelMA MS for MMS fabrication. MPN modification was performed using tannic acid (TA) and strontium chloride hexahydrate (SrCl_2_·6H_2_O) at equal mass concentrations (0.125, 0.25, 0.5, and 1 mg/mL). Briefly, 2 mL of TA solution was added to 1 mL of fully hydrated GelMA MS and mixed thoroughly. The mixture was incubated on a shaker at room temperature for 5 min. After removal of the supernatant, 1 mL of strontium chloride solution was added. An alkaline buffer was then introduced with gentle shaking to adjust the pH to 8.0. The mixture was incubated on a shaker for 20 min. After removal of the supernatant, another 1 mL of TA solution was added, the pH was readjusted to 8.0, and the mixture was incubated for an additional 20 min. Finally, the synthesized MMS were washed three times with deionized water. Using similar microfluidic methods reported previously, HAMA‐, chondroitin sulfate (CS)‐, and chitosan‐based microgels were prepared as controls [88, 91]. These microgels were further modified with MPN coatings using polyphenols, including TA, TP, and EGCG, following the same protocol. This approach was used to evaluate the universality of MPNs as functional surface coatings for different microgel systems.

TRP was loaded onto MMS using a low‐temperature incubation method to construct the MMS@TRP delivery system. The optimal TRP‐to‐MMS loading ratio was determined as follows. Briefly, 500 µL of Cy5‐labeled TRP solution was mixed with 500 µL of MMS prepared using different TA/Sr^2+^ MPN concentrations. The mixtures were incubated on a shaker at 4°C for 6 h, followed by centrifugation and three washes with deionized water. The supernatants and wash solutions were collected, and unbound TRP was quantified using a BioTek Synergy H1 microplate reader. These data were used to determine the optimal TA/Sr^2+^ modification concentration for TRP loading. Additionally, different volumes of TRP (1 µm) were mixed with 1 mL of MMS to evaluate loading efficiency.

Bright‐field microscopy was used to observe morphological and color changes in the microgels after MPN modification and TRP loading. Scanning electron microscopy (SEM) was performed to characterize the morphology and surface structures of GelMA MS, MMS, and MMS@TRP. SEM elemental mapping was conducted to analyze changes in surface elemental composition following MPN modification and TRP loading. Laser scanning confocal microscopy (LSCM) was used to verify successful loading of TRP containing FAM‐labeled miR‐21 inhibitor and Cy5‐labeled tFNAs onto MMS. Fourier‐transform infrared spectroscopy (FTIR) and X‐ray photoelectron spectroscopy (XPS) were further applied to analyze molecular structural and elemental changes during MMS@TRP fabrication.

For the in vitro degradation assay, MMS@TRP microgels were separately immersed in 2 µg mL^−^^1^ collagenase II solution or 10% FBS‐containing medium and incubated at 37°C on a shaker at 50 rpm. At weekly intervals, the morphological changes of the microgels were observed by microscopy, and the degradation rate was quantitatively determined by measuring the dry weight of the remaining microgels. The degradation rate was calculated as (W_0_ − Wt)/W_0_ × 100%, where W_0_ and Wt represent the initial dry weight and the remaining dry weight at each time point, respectively.

### 4.10 Evaluation of TA‐Mediated Protection Against Nuclease Degradation

Molecular docking was performed to evaluate the binding ability and affinity of TA toward DNase I and RNase A, with particular focus on the spatial relationship between the TA‐binding sites and the catalytic sites of the nucleases responsible for DNA and RNA degradation. A 10% FBS‐containing medium was used to mimic a nuclease‐rich degradation environment. PAGE analysis was performed to evaluate the protective effects of TA against nuclease‐mediated degradation of tFNAs and the miR‐21 inhibitor.

To evaluate the protective effect of TA on tFNAs, TA at concentrations of 0, 5, 10, 20, 50, 100, and 200 µg/mL was added to 10% FBS‐containing medium and incubated at room temperature for 1 h. Subsequently, tFNAs (200 nM) were added, and the mixtures were incubated at 37°C for 24 h, followed by PAGE analysis. Additional experiments were performed to determine whether TA at a cell‐safe concentration (40 µg/mL) could protect TRP against degradation. TRP samples were incubated in 10% FBS‐containing medium at 37°C for 6, 12, 24, 48, 72, and 96 h in the presence or absence of TA, followed by PAGE analysis. The protective effect of TA on the miR‐21 inhibitor against nuclease‐mediated degradation was evaluated using the same experimental procedure. Briefly, the miR‐21 inhibitor was incubated in 10% FBS‐containing medium with or without TA, and its integrity at the indicated time points was subsequently evaluated by PAGE.

To investigate the protective delivery effect of MMS on TRP, MMS@TRP was prepared using Cy5‐labeled tFNAs and FAM‐labeled miR‐21 inhibitor. MMS@TRP was mixed with 10% FBS medium at a 1:2 volume ratio and incubated at 37°C for 1, 3, 5, and 7 days. Fluorescence colocalization analysis was subsequently performed to evaluate TRP release behavior and the protective effect of MMS against DNase‐mediated TRP degradation.

### 4.11 Assessment of Microgel System Biocompatibility

The biocompatibility of the microgel systems (MS, MMS, and MMS@TRP) was evaluated by mixing 1 mL of each microgel with 2 mL of culture medium. NPCs were seeded in 96‐well plates (2 × 10^3^ cells per well) and cultured until reaching 50% confluence. Cells were then incubated with medium containing the respective microgels. Cell viability was assessed after 24 and 48 h using the CCK‐8 assay and live‐dead staining. For the CCK‐8 assay, the supernatant and microgels were removed, and cells were washed three times with fresh medium. Cells were subsequently incubated with 100 µL complete medium containing 10% CCK‐8 reagent at 37°C for 2 h. The optical density (OD) at 450 nm was recorded using a SYNERGY H1 microplate reader. For live‐dead staining, a solution containing 10 µL propidium iodide (PI) and 5 µL calcein AM in 5 mL PBS was prepared. Cells were stained at 37°C for 15 min, rinsed with PBS, and imaged using a Revvity High Content Cell Imaging Analyzer.

### 4.12 Analysis of TRP and TA Release

Release kinetics of TRP and TA from MMS@TRP were evaluated by placing samples (1 mL, Cy3‐labeled TRP) into microfiltration membranes (0.45 µm pore size; n = 3) submerged in different release media (physiological conditions, pH 7.4, and acidic conditions, pH 6.2) at 37°C. Samples were collected at predetermined intervals (12, 24, 72, 120, and 168 h). TRP release was quantified using a BioTek Synergy H1 microplate reader. TA release was measured separately at intervals of 1, 3, 7, 14, and 21 days using a UV–Vis spectrophotometer.

### 4.13 Retention of miR‐21 Inhibitor After In Vivo Delivery

The in vivo retention of the miR‐21 inhibitor was assessed using Cy3‐labeled constructs in a rat model. Three formulations (20 µL each: miR‐21 inhibitor (630 nm), TRP (630 nm), or MMS@TRP) were injected into intervertebral discs at Co 2/3, 4/5, and 6/7. Fluorescent retention was monitored at 1, 2, and 4 days post‐injection using an IVIS Spectrum imaging system. Major organs, including the heart, liver, spleen, lungs, kidneys, and brain, were collected at 1, 2, and 4 days after MMS@TRP administration for ex vivo fluorescence imaging to assess potential systemic distribution.

### 4.14 Targeted Delivery of TRP to Pericytes via MMS@TRP

To evaluate targeted TRP delivery to pericytes via MMS@TRP, Cy5‐labeled TRP was employed. Pericytes pretreated with dNPC supernatants were exposed to TRP (200 nm) or MMS@TRP (320 µL/mL) for 24 h. Fluorescence detection compared intracellular uptake efficiency between groups. Before application, MMS@TRP was pre‐incubated in medium at pH 6.5 for 12 h, then adjusted to pH 7.4.

To further assess in vivo targeted delivery to pericytes in degenerated intervertebral discs (IVDs), Cy5‐labeled TR and TRP were used to prepare MMS@TR and MMS@TRP. Four weeks after establishing rat IVD degeneration, MMS@TR and MMS@TRP (20 µL) were injected into discs at levels Co3/4 and Co5/6, respectively (n = 3). After 12 h, caudal vertebrae were harvested, and frozen sections were prepared [58]. Immunofluorescence staining for PDGFRB and fluorescence colocalization analysis with Cy5 evaluated the effectiveness of MMS@TRP for targeted pericyte delivery within degenerated IVDs.

### 4.15 In Vitro Treatment of Pericytes

To evaluate the therapeutic effects of the MMS@TRP system on pericytes, five experimental groups were established: Control, NC, TRP, MMS, and MMS@TRP (n = 3 or 6 per group). Before treatment, the Control group was incubated with conditioned medium from normal NPCs for 24 h, whereas the other groups were treated with conditioned medium from degenerated NPCs (dNPCs) for 24 h. Subsequently, cells were treated with TRP (200 nm), MMS (320 µL/mL), or MMS@TRP (320 µL/mL) (n = 3 per treatment group).

Cell proliferation was assessed using the CCK‐8 assay at 24 and 48 h after treatment. Cell migration was evaluated using a Transwell assay at 48 h. Western blotting and immunofluorescence staining were performed to detect the expression of Smad7, Smad2/3, p‐Smad2/3, Col1, and α‐SMA. Furthermore, transcriptome sequencing was conducted to investigate the molecular mechanisms underlying the therapeutic effects of MMS@TRP on pericytes.

### 4.16 In Vitro Treatment of NPCs

NPCs were treated with 50 µm TBHP for 24 h, followed by treatment with TRP (200 nm), MMS (320 µL/mL), or MMS@TRP (320 µL/mL) (n = 3 or 6). Cell viability was evaluated using the CCK‐8 assay and live/dead staining. ROS‐scavenging activity was assessed using ABTS, PTIO, and DPPH radical scavenging assays, as well as DCFH‐DA staining [92]. Mitochondrial membrane potential (ΔΨm) and mitochondrial ROS (mtROS) levels were analyzed using JC‐1 staining and MitoSOX staining, respectively [93]. Anti‐inflammatory activity was evaluated by measuring the expression of IL‐1β, IL‐10, and TNF‐α using RT‐qPCR, western blotting, and immunofluorescence staining. ECM metabolism was further assessed by analyzing anabolic markers (Col2 and Acan) and catabolic enzymes (MMP13 and ADAMTS5) using the same molecular biology techniques.

### 4.17 In Vitro Treatment of Macrophages

RAW264.7 macrophages were stimulated with LPS (1 µg/mL) for 24 h to induce an inflammatory response, followed by treatment with TRP (200 nm), MMS (320 µL/mL), or MMS@TRP (320 µL/mL) (n = 3). Immunofluorescence staining was performed to evaluate the expression of TNF‐α and IL‐10.

### 4.18 In Vivo Analysis of IVDD Treatment

Seven days after IVDD model establishment, 20 µL of PBS, TRP (630 nm), MMS, or MMS@TRP was injected into the IVD. Disc morphology and degeneration were evaluated at 4 and 8 weeks post‐treatment. X‐ray imaging was used to calculate the disc height index (DHI), and MRI was performed to assess the degeneration grade [88]. At 8 weeks after treatment, an independent set of vertebra–disc–vertebra motion segments was harvested from each group for biomechanical evaluation (n = 5). Axial compression testing was performed using a universal testing machine at a displacement rate of 1.0 mm/min until a strain of 5% was reached. Stress–strain curves were generated to evaluate the compressive mechanical response of the IVD. In parallel, separate samples collected for histological evaluation (n = 5) were fixed in 4% paraformaldehyde, decalcified in 10% EDTA for 2–4 weeks, embedded in paraffin, and sectioned at 5 µm. H&E and Safranin‐O/Fast Green staining were performed to evaluate IVD morphology, proteoglycan distribution, and histological degeneration scores [90]. Immunofluorescence staining was performed to assess fibrosis (Col1), inflammation (TNF‐α), and ECM metabolism‐related proteins (Col2, Acan, MMP13, and ADAMTS5). To further evaluate the therapeutic contribution of PDGFRB‐mediated pericyte targeting, an additional in vivo comparison between MMS@TR and MMS@TRP was performed. At 8 weeks after treatment, IVD tissues were harvested for H&E and Safranin‐O/Fast Green staining, histological scoring, and immunohistochemical analysis of Col1 and Col2 expression (n = 5).

### 4.19 Systemic Biosafety Evaluation of MMS@TRP

To evaluate the systemic biosafety of the MMS@TRP platform, a hemolysis assay was performed to assess blood compatibility. Additionally, potential systemic toxicity was evaluated by H&E staining of major organs, including the heart, liver, spleen, lungs, and kidneys, collected at 8 weeks after treatment.

### 4.20 Molecular Biological Detection

#### 4.20.1 RT‐qPCR

Total RNA was extracted from cells and reverse‐transcribed into cDNA. Gene‐specific primers were designed, and expression levels were quantified using SYBR Green‐based qPCR. Relative mRNA expression levels were calculated using the 2^−ΔΔCt^ method and normalized to GAPDH as the internal control.

#### 4.20.2 Western Blot Analysis

Proteins were extracted using RIPA lysis buffer and quantified using a BCA assay. Protein samples were separated by SDS‐PAGE and transferred onto PVDF membranes. After overnight incubation with primary antibodies, membranes were incubated with HRP‐conjugated secondary antibodies. Protein bands were visualized using enhanced chemiluminescence (ECL).

#### 4.20.3 Cell Immunofluorescence

Cells were fixed with 4% paraformaldehyde, followed by permeabilization and blocking using QuickBlock Blocking Buffer. Cells were incubated with primary antibodies overnight at 4°C and then incubated with Alexa Fluor‐conjugated secondary antibodies in the dark. Cytoskeletal structures were stained with phalloidin, and nuclei were counterstained with DAPI. Fluorescence intensity and localization of target proteins were visualized using a fluorescence microscope or a Revvity High Content Cell Imaging Analyzer.

#### 4.20.4 Flow Cytometry Analysis

For flow cytometry analysis, pericytes were washed with PBS, digested with trypsin (without EDTA), centrifuged, and collected. The cells were then resuspended in cold PBS and analyzed using flow cytometry.

#### 4.20.5 ELISA

The collected supernatant was centrifuged at 300 g for 10 min to remove dead cells and debris. The TGFβ1 levels in the supernatant were analyzed using the ELISA kit (Jonlnbio, Shanghai, China) according to the manufacturer's instructions.

### 4.21 Statistical Analyses

All statistical analyses were performed using GraphPad Prism 9.0 software. Quantitative data are presented as mean ± standard deviation (SD). Normality and homogeneity of variance were assessed where applicable. Comparisons between two groups were performed using an unpaired two‐tailed Student's t‐test or Mann–Whitney U test, as appropriate. Comparisons among multiple groups were performed using one‐way or two‐way ANOVA followed by Tukey's multiple‐comparisons test. Statistical significance was defined as *P* < 0.05 (^*^), *P* < 0.01 (^**^), *P* < 0.001 (^***^), and ns (not significant).

## Author Contributions

**Yuheng Liu**: methodology, formal analysis. **Yu Wang**: methodology, funding acquisition. **Dengbo Yao**: visualization, data curation. **Weiqiang Lan**: visualization, software. **Mei Zhang**: funding acquisition, methodology, supervision. **Chen Fan**: investigation, data curation. **Chuan Guo**: supervision, funding acquisition, software. **Zhen Zhao**: conceptualization, methodology, formal analysis, writing – original draft, writing – review and editing. **Dejun Zhong**: funding acquisition, formal analysis. **Qingquan Kon**: funding acquisition, conceptualization, methodology, supervision. **Fei Ma**: conceptualization, methodology, data curation, writing – original draft, writing – review and editing. **Yunfeng Lin**: conceptualization, funding acquisition, supervision, writing – review and editing, resources. **Yao He**: software, data curation.

## Conflicts of Interest

The authors declare no conflicts of interest.

## Supporting information




**Supporting File**: advs77987‐sup‐0001‐SuppMat.docx.

## Data Availability

The data that support the findings of this study are available from the corresponding author upon reasonable request.
