# Supplementary material for: Versatile Microgel Platform for Intervertebral Disc Degeneration Therapy: Targeting Pericyte‐Mediated Fibrosis and Protecting Nucleus Pulposus Cells
Source: Adv Sci (Weinh). 2026 Sep 30:e77987. Online ahead of print. doi: 10.1002/advs.77987 (PMC13625588; doi:10.1002/advs.77987)
Supplement: Supplementary file 1 [file ADVS-9999-e77987-s001.docx]

**Supporting Information**

**Versatile Microgel Platform for Intervertebral Disc Degeneration Therapy: Targeting Pericyte-Mediated Fibrosis and Protecting Nucleus Pulposus Cells**

*Fei Ma**^1,2#^, Zhen Zhao^1#^, Chuan Guo^1^, Yuheng Liu^1^, Yu Wang^1^, Dengbo Yao^1^, Weiqiang Lan^1^, Dejun Zhong^2^, Chen Fan^4^, Yao He^5^, Qingquan Kong^1,3^*, Yunfeng Lin^3^**

1, Department of Orthopedic Surgery and Orthopedic Research Institute, West China Hospital, Sichuan University, Chengdu 610041, P. R. China.

2, Department of Orthopedics, The Affiliated Hospital of Southwest Medical University, Luzhou 646000, P. R. China.

3, State Key Laboratory of Oral Diseases, National Center for Stomatology, National Clinical Research Center for Oral Diseases, West China Hospital of Stomatology, Sichuan University, Chengdu 610041, P. R. China.

4, Department of Orthopedics Surgery, Hospital of Chengdu Office of People’s Government of Tibetan Autonomous Region, Chengdu 610041, P. R. China.

5, Macao Translational Medicine Center, Macau University of Science and Technology, Taipa 999078, P. R. China.

Author list:

Fei Ma. PhD. First author.

Department of Orthopedic Surgery and Orthopedic Research Institute, West China Hospital, Sichuan University, Chengdu 610041, P. R. China.

Department of Orthopedics, The Affiliated Hospital of Southwest Medical University, Luzhou 646000, P. R. China.

E-mail address:396973165@qq.com

Zhen Zhao. PhD. Author 2

Department of Orthopedic Surgery and Orthopedic Research Institute, West China Hospital, Sichuan University, Chengdu 610041, P. R. China.

E-mail address: zhaozhen101013@163.com

Chuan Guo. PhD. Author 3

Department of Orthopedic Surgery and Orthopedic Research Institute, West China Hospital, Sichuan University, Chengdu 610041, P. R. China.

E-mail address: guochuan@wchscu.cn

Yuheng Liu. PhD. Author 4

Department of Orthopedic Surgery and Orthopedic Research Institute, West China Hospital, Sichuan University, Chengdu 610041, P. R. China.

E-mail address: llllllyh@126.com

Yu Wang. PhD. Author 5

Department of Orthopedic Surgery and Orthopedic Research Institute, West China Hospital, Sichuan University, Chengdu 610041, P. R. China.

E-mail address: [wangyu2019@wchscu.cn](mailto:wangyu2019@wchscu.cn)

Dengbo Yao. PhD. Author 6

Department of Orthopedic Surgery and Orthopedic Research Institute, West China Hospital, Sichuan University, Chengdu 610041, P. R. China.

E-mail address: yaodb@wchscu.edu.cn

Weiqiang Lan. PhD. Author 7

Department of Orthopedic Surgery and Orthopedic Research Institute, West China Hospital, Sichuan University, Chengdu 610041, P. R. China.

E-mail address: lanwq0911@163.com

Dejun Zhong. PhD. Author 8

Department of Orthopedics, The Affiliated Hospital of Southwest Medical University, Luzhou 646000, P. R. China.

E-mail address: zdj_1974@163.com

Chen Fan. Author 9

Department of Orthopedics Surgery, Hospital of Chengdu Office of People’s Government of Tibetan Autonomous Region, Chengdu 610041, P. R. China.

E-mail address: 755130667@qq.com

Yao He. PhD. Author 10

Macao Translational Medicine Center, Macau University of Science and Technology, Taipa 999078, P. R. China.

E-mail address: yaohe@must.edu.mo

Qingquan Kong. Prof. Corresponding Author^*^

Department of Orthopedic Surgery and Orthopedic Research Institute, West China Hospital, Sichuan University, Chengdu 610041, P. R. China.

Department of Orthopedics Surgery, Hospital of Chengdu Office of People's Government of Tibetan Autonomous Region, Chengdu 610041, P. R. China.

E-mail address: kqqspine@126.com

Yunfeng Lin. Prof. Corresponding Author*

State Key Laboratory of Oral Diseases, National Center for Stomatology, National Clinical Research Center for Oral Diseases, West China Hospital of Stomatology, Sichuan University, Chengdu 610041, P. R. China.

E-mail address: [yunfenglin@scu.edu.cn](mailto:yunfenglin@scu.edu.cn)

1. **Results**

**Figure S1.** (A, B) Volcano plots of DEGs in pericytes identified by single-cell RNA sequencing. (C) Quantitative analysis of Col1 immunofluorescence in healthy and degenerated rat intervertebral discs (n=3). (D) Quantitative analysis of Col1 immunofluorescence in rat IVDs from the degeneration model group and the pericyte transplantation group (n=3). Data are presented as mean ± SD. Error bars represent SD. Statistics: unpaired two-tailed Student’s t-test was used. Significance: ** p < 0.01.

**Figure S2.** (A) CCK-8 assay showing the effects of conditioned media from non-degenerated and degenerated NPCs on pericyte proliferation (n=6). (B) Quantitative immunofluorescence analysis of α-SMA and Col1 expression in pericytes treated with dNPC-conditioned medium (n=3). (C) PCA of transcriptomic sequencing data. (D) Heatmap of DEGs identified by transcriptomic sequencing. (E) GSEA enrichment analysis of the PI3K-Akt signaling pathway. (F) Quantitative western blot analysis of TGFβ1 expression in NPCs from the control and TBHP-induced degeneration groups (n=3). (G) Quantitative immunofluorescence analysis of TGFβ1 expression in mildly and severely degenerated human NP tissues (n=3). (H) Quantitative immunofluorescence analysis of TGFβ1 expression in normal and degenerated rat IVDs (n=3). (I) Immunofluorescence staining of Col1 and α-SMA expression in pericytes treated with dNPC-conditioned medium or dNPC-conditioned medium + TGFβ1 antibodies. Data are presented as mean ± SD. Error bars represent SD. Statistics: One-way ANOVA with Tukey’s multiple-comparisons test was used for panels (A,B); unpaired two-tailed Student’s t-test for panels (F-H). Significance: ** p < 0.01, *** p < 0.001.

**Figure S3.** **TGFβ1 is a major factor mediating dNPC-induced pericyte fibrotic transformation.** (A-E) Western blot and quantitative analyses showing the effects of recombinant TGFβ1 and SB-431542 on the expression of Smad7, p-Smad2/3, Smad2/3, α-SMA, and Col1 in pericytes (n=3). (F-J) Western blot and quantitative analyses showing the effects of dNPC-conditioned medium and SB-431542 on the expression of Smad7, p-Smad2/3, Smad2/3, α-SMA, and Col1 in pericytes (n=3). Data are presented as mean ± SD. Error bars represent SD. Statistics: One-way ANOVA with Tukey’s multiple-comparisons test was used. Significance: * p < 0.05, ** p < 0.01, *** p < 0.001.

**Figure S4.** Functional validation of the contribution of PI3K-Akt signaling to dNPC-induced alterations in pericyte behavior. (A, B) Western blot and quantitative analyses showing the effects of dNPC-conditioned medium and LY294002 on p-Akt, Akt, α-SMA, and Col1 expression in pericytes (n=3). (C) CCK-8 assay evaluating the effects of dNPC-conditioned medium and LY294002 on pericyte proliferation (n=6). (D) Transwell assay evaluating the effects of dNPC-conditioned medium and LY294002 on pericyte migration. Data are presented as mean ± SD. Error bars represent SD. Statistics: One-way ANOVA with Tukey’s multiple-comparisons test was used. Significance: * p < 0.05, ** p < 0.01, *** p < 0.001; ns, not significant.

**Figure S5.** (A) Immunofluorescence staining of PDGFRB and Smad7 expression in PDGFRB-positive cells from mildly and severely degenerated human IVD tissues. (B) Immunofluorescence staining of PDGFRB and Smad7 expression in PDGFRB-positive cells from normal and degenerated rat IVDs. (C) Overlap analysis of miRNAs targeting Smad7 predicted by miRDB, TargetScan, and miRTargetLink.

**Figure S6.** Dual-luciferase reporter assay. (A) Sequence alignment demonstrating complementarity between miR-21 and binding sites in Smad7 3′-UTRs. (B, C) Dual-luciferase reporter assay analyzing miR-21 binding to wild-type (WT) or mutated Smad7 3′-UTRs (n=3). Data are presented as mean ± SD. Error bars represent SD. Statistics: unpaired two-tailed Student’s t-test was used. Significance: ** p < 0.01; ns, not significant.

**Figure S7.** Smad7 knockdown attenuates the anti-fibrotic effects of miR-21 inhibition in pericytes. (A) Western blot analysis of Smad7, p-Smad2/3, Smad2/3, α-SMA, and Col1 expression in pericytes following miR-21 inhibition and siRNA-mediated Smad7 knockdown. (B-E) Quantitative analyses of Smad7, p-Smad2/3, Smad2/3, α-SMA, and Col1 expression. Data are presented as mean ± SD. Error bars represent SD. Statistics: one-way ANOVA with Tukey’s multiple-comparisons test was used. Significance: * p < 0.05, ** p < 0.01.

**Figure S8.** (A) CCK-8 assay evaluating NPC proliferation after treatment with different concentrations of TRP for 24 and 48 h (n=6). (B) Immunofluorescence staining of Smad7 expression in pericytes treated with different concentrations of TRP. (C) Quantitative immunofluorescence analysis of Smad7 expression (n=3). (D) Quantitative immunofluorescence analysis of PDGFRB expression in pericytes from the control and dNPC-conditioned medium groups (n=3). (E) Quantitative western blot analysis of PDGFRB expression in pericytes from the control and dNPC-conditioned medium groups (n=3). (F) Immunofluorescence staining of Smad7 expression in pericytes treated with dNPC-conditioned medium following TRP treatment. (G) Immunofluorescence staining of Smad2/3 and p-Smad2/3 expression in pericytes treated with dNPC-conditioned medium following TRP treatment. (H) Immunofluorescence staining of Col1 and α-SMA expression in pericytes treated with dNPC-conditioned medium following TRP treatment. (I) Quantitative immunofluorescence analysis of Smad7 expression (n=3). (J) Quantitative immunofluorescence analysis of Smad2/3 and p-Smad2/3 expression (n=3). (K) Quantitative immunofluorescence analysis of Col1 and α-SMA expression (n=3). Data are presented as mean ± SD. Error bars represent SD. Statistics: two-way ANOVA with Tukey’s multiple-comparisons test was used for panel (A); one-way ANOVA with Tukey’s multiple-comparisons test was used for panels (C,I,J,K); unpaired two-tailed Student’s t-test was used for panels (D,E). Significance: * p < 0.05, ** p < 0.01; ns, not significant.

**Figure S9.** Comparison of TRP uptake among different cell types. (A) Representative confocal fluorescence images of Cy5-TRP uptake by pericytes, nucleus pulposus cells (NPCs), and macrophages at 2 and 6 h after incubation. (B,C) Quantitative analysis of intracellular Cy5 fluorescence intensity in the three cell types at 2 and 6 h, respectively (n=3). Data are presented as mean ± SD. Error bars represent SD. Statistics: One-way ANOVA with Tukey’s multiple-comparisons test was used for panels (B,C). Statistical significance is indicated as *p < 0.05 and **p < 0.01.

**Figure S10.** (A) ¹H-NMR spectra of Gel and GelMA. (B) Appearance and light microscopy images showing coloration resulting from polyphenol-MPN modifications on microspheres composed of different materials. (C) DPPH scavenging assay demonstrating the ROS-scavenging capacity of GelMA microspheres (MS) modified with various concentrations of TA/Sr^2+^ (n=3). Data are presented as mean ± SD. Error bars represent SD.

**Figure S11.** In vitro degradation of MMS@TRP. (A) Representative microscopic images showing the degradation of MMS@TRP at different time points. (B) Quantitative degradation profile of MMS@TRP over time (n=3). Data are presented as mean ± SD. Error bars represent SD.

**Figure S12.** Molecular docking and PAGE analysis. (A) Molecular docking and colocalization analyses of TA and RNase A. (B) PAGE analysis of TA inhibition of miR-21 inhibitor degradation in 10% FBS over time.

**Figure S13.** (A) CCK-8 assay evaluating the biocompatibility of MS, MMS, and MMS@TRP with NPCs (n = 6). (B) Live/dead staining assessing the biocompatibility of MS, MMS, and MMS@TRP with NPCs (n=3). Data are presented as mean ± SD. Error bars represent SD. Statistics: two-way ANOVA with Tukey’s multiple-comparisons test was used for panel (A). ns, not significant.

**Figure S14.** Fluorescence imaging of major organs following intradiscal injection of MMS@TRP. White arrows indicate the fluorescence signal at the rat tail injection site.

**Figure S15.** Fluorescence colocalization analysis demonstrating uptake of released Cy5-TR by PDGFRB-positive pericytes in the outer AF, inner AF, and CE regions following MMS@TR injection.

**Figure S16.** (A) CCK-8 assay evaluating pericyte proliferation after treatment with MMS@TRP at 24 and 48 h (n = 6). (B) GSEA enrichment analysis results for the PI3K-Akt signaling pathway. Data are presented as mean ± SD. Error bars represent SD. Statistics: two-way ANOVA with Tukey’s multiple-comparisons test was used for panel (A).

**Figure S17.** Protective effects of MMS@TRP on NPCs. (A) CCK-8 assay showing NPC proliferation following MMS@TRP treatment under TBHP-induced conditions (n=6). (B) Live/dead staining demonstrating the protective effects of MMS@TRP against TBHP-induced NPC death. (C-E) Immunofluorescence staining showing the impact of MMS@TRP on the expression of inflammatory factors (IL-10, TNF-α, and IL-1β) in TBHP-treated NPCs. (F-H) Quantitative analysis of immunofluorescence images illustrating the effects of MMS@TRP treatment on the inflammatory markers IL-10, TNF-α, and IL-1β in TBHP-treated NPCs (n=3). Data are presented as mean ± SD. Error bars represent SD. Statistics: two-way ANOVA with Tukey’s multiple-comparisons test was used for panel (A); one-way ANOVA with Tukey’s multiple-comparisons test was used for panels (F-H). Significance: * p < 0.05, ** p < 0.01, ***p < 0.001.

**Figure S18.** MMS@TRP regulates inflammatory cytokine expression in macrophages. (A, B) Representative immunofluorescence images and quantitative analysis of IL-10 and TNF-α expression in RAW264.7 macrophages following the indicated treatments. Data are presented as mean ± SD. Error bars represent SD. Statistics: One-way ANOVA with Tukey’s multiple-comparisons test was used for panel (B). Significance: **p < 0.01.

**Figure S19.** Evaluation of the in vivo therapeutic efficacy of MMS@TRP in a rat IVDD model. (A, B) Immunofluorescence staining of ECM components (Acan and Col2) at 8 weeks. (C-E) Immunofluorescence staining of ECM catabolic enzymes (MMP13 and ADAMTS5) and the inflammatory marker TNF-α at 8 weeks.

**Figure S20.** In vivo comparison of therapeutic efficacy between MMS@TRP and MMS@TR. (A) Representative H&E and Safranin O/Fast Green staining images at 8 weeks after treatment. (B) Histological scores (n = 5). (C-F) Immunohistochemical analysis and quantification of Col1 and Col2 expression (n = 5). Data are presented as mean ± SD. Error bars represent SD. Statistics: The Mann–Whitney U test was used for panel (B); unpaired two-tailed Student’s t-test was used for panels (E, F). Significance: * p < 0.05; ns, not significant.

**Figure S21. Biomechanical evaluation of intervertebral discs after MMS@TRP treatment.** (A) Schematic of axial compression testing of vertebra–disc–vertebra motion segments. (B) Stress–strain curves of different treatment groups.

**Figure S22. Systemic biosafety evaluation of MMS@TRP treatment.** (A) Hemolysis assay (n=3). (B) H&E staining of major organs (heart, liver, spleen, lungs, and kidneys). Data are presented as mean ± SD. Error bars represent SD.

**Table S1 Primer sequences**

|  | **Base sequence (5′ → 3′)** |
| --- | --- |
| **S2-P** | GGTTGTTGGGTTACATGCGAGGGTCCAATACCGACGATTACAGCTTGCTACACGATTCAGACTTAGGAATGTTCG |
| **S3-R** | TAGCTTATCAGACTGATGTTGATTACTACTATGGCGGGTGATAAAACGTGTAGCAAGCTGTAATCGACGGGAAGAGCATGCCCATCC |
| **miR-21 inhibitor** | UCAACAUCAGUCUGAUAAGCUA |
| **pPB** | CCCAACAACC-CSRNLIDC |
